# Cortical connectivity in the face of congenital structural changes—A case of homozygous *LAMC3* mutation

**DOI:** 10.1002/brb3.2241

**Published:** 2021-06-14

**Authors:** Pinar Demirayak, Kader Karli Oguz, Fatma Seyhun Ustun, Buse Merve Urgen, Yasemin Topac, Irtiza Gilani, Tulay Kansu, Serap Saygi, Tayfun Ozcelik, Huseyin Boyaci, Katja Doerschner

**Affiliations:** ^1^ Department of Neurobiology University of Alabama at Birmingham Birmingham Alabama USA; ^2^ A.S. Brain Research Center and National Magnetic Resonance Center Bilkent University Ankara Turkey; ^3^ Department of Radiology Hacettepe University Ankara Turkey; ^4^ Neuroscience Program Bilkent University Ankara Turkey; ^5^ Department of Neurology Hacettepe University Ankara Turkey; ^6^ Department of Molecular Biology and Genetics Bilkent University Ankara Turkey; ^7^ Department of Psychology Bilkent University Ankara Turkey; ^8^ Department of Psychology JL Giessen University Giessen Germany

**Keywords:** diffusion tensor imaging, functional connectivity, LAMC3, probabilistic tracktography, resting state, structural connectivity

## Abstract

The homozygous *LAMC3* gene mutation is associated with severe bilateral smoothening and thickening of the lateral occipital cortex . Despite this and further significant changes in gray matter structure, a patient harboring this mutation exhibited a range of remarkably intact perceptual abilities . One possible explanation of this perceptual sparing could be that the white matter structural integrity and functional connectivity in relevant pathways remained intact. To test this idea, we used diffusion tensor and functional magnetic resonance imaging to investigate functional connectivity in resting‐state networks in major structural pathways involved in object perception and visual attention and corresponding microstructural integrity in a patient with homozygous *LAMC3* mutation and sex, age, education, and socioeconomically matched healthy control group. White matter microstructural integrity results indicated widespread disruptions in both intra‐ and interhemispheric structural connections except inferior longitudinal fasciculus. With a few exceptions, the functional connectivity between the patient's adjacent gray matter regions of major white matter tracts of interest was conserved. In addition, functional localizers for face, object, and place areas showed similar results with a representative control, providing an explanation for the patient's intact face, place, and object recognition abilities. To generalize this finding, we also compared functional connectivity between early visual areas and face, place, and object category‐selective areas, and we found that the functional connectivity of the patient was not different from the control group. Overall, our results provided complementary information about the effects of *LAMC3* gene mutation on the human brain including intact temporo‐occipital structural and functional connectivity that are compatible with preserved perceptual abilities.

## Graphical abstract

While structural results predominantly point to widespread disruptions in intrahemispheric and interhemispheric connectivity, functional connectivity seemed to have been conserved, especially between left inferior temporo‐occipital areas including the inferior temporal gyrus and the lateral occipital cortex. These functional results could provide an explanation for the patient's intact face and object recognition abilities. Taken together our findings serve as a striking example of the brain's ability to maintain normal function in the face of gross congenital structural changes, and that the relation between cortical structure and function is not necessarily isomorphic.

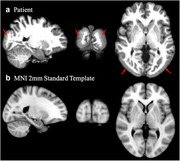

## 1 INTRODUCTION

**FIGURE 1:**
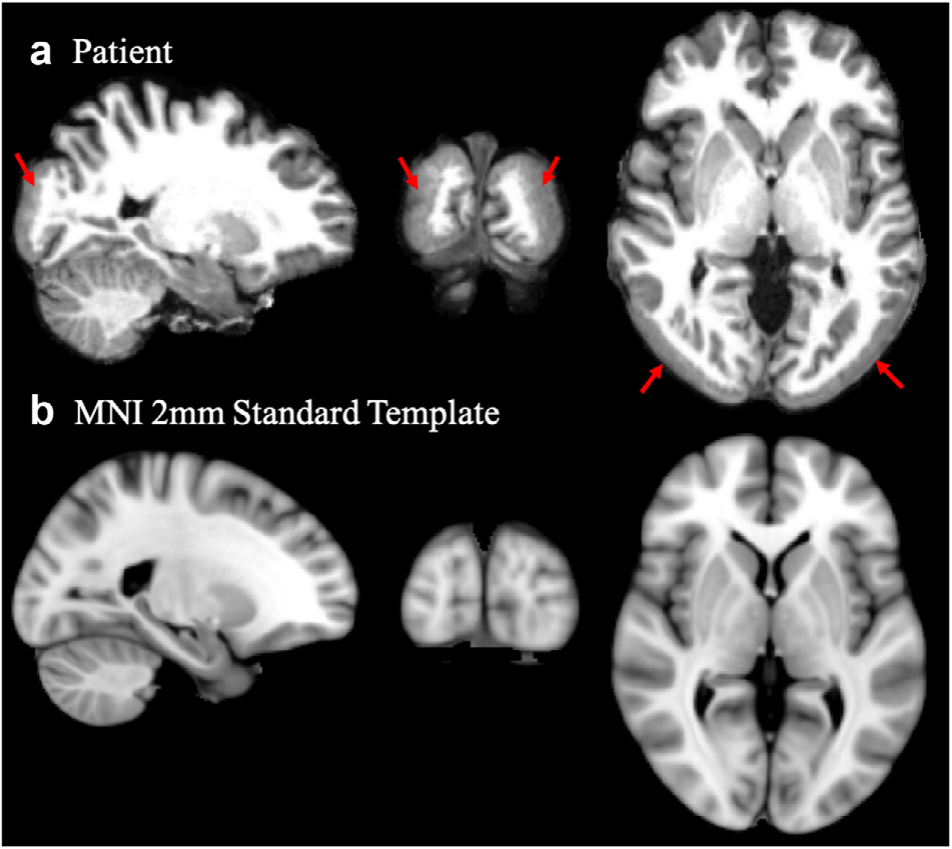
T1‐weighted images of the patient's brain (a) and corresponding views of the MNI 2 mm standard template (b). Shown are sagittal, coronal, and transversal views (from left to right). Areas overlaid with a red color (and a dotted outline) highlight the pachygyric regions in the occipital lobes of the patient, that is, the cortical areas that show massive structural abnormalities compared to the average brain (below)

Patient NG 367‐1, (Barak et al., 2011) is homozygous for a single mutation in the *LAMC3* gene and shows severe bilateral smoothening and thickening (>8 mm) of the lateral occipital lobes (Figure 1). Urgen et al. (2019) found that the cortical structural changes in this patient's brain involve regions beyond the occipital lobes and primarily those that are part of the dorsal frontoparietal and the ventral attention network. They argued that these anatomical changes might explain why the patient showed pronounced behavioral deficits in visuospatial attention (Urgen et al., 2019). Strikingly, however, her face, place, and object recognition remain intact, *despite* significantly thick gray matter structure in the lateral occipital cortex (LOC), inferior temporal (IT) gyrus, parahippocampal place area (PPA), and fusiform gyrus (FFA) that are shown to be associated with object and face processing previously (Grill‐Spector et al., 2001; Kanwisher & Yovel, 2006 ), and Urgen and her colleagues (2019) speculated that the preserved visual abilities of the patient might be mediated by increases in efficiency of neural processing (Li et al., 2009).

This speculation was based on an observed increase in white matter volume (WMV) in several cortical regions, including the IT and LOC (Urgen et al., 2019). However, WMV is not necessarily an indicator of well‐organized fibers, instead, fiber organization indices such as fractional anisotropy (FA) (Hulst et al., 2013; Klingberg et al., 2000; Nagy et al., 2004; Schmithorst et al., 2002) and measures of diffusivity (Penke et al., 2010; Qiu et al., 2008; Vernooij et al., 2009) tend to be correlated with cognitive functions. Barak et al. (2011) found decreases in FA in white matter pathways and a total lack of short U‐ fibers in the lateral occipital region of this patient. However, object and face perception are not only subserved by single cortical regions (such as FFA, PPA, or LOC) but also involve information flow in distributed pathways (Schwarzlose et al., 2008).

It is generally assumed that functional connectivity reflects the structural connectivity (white matter axonal connections between brain regions) (Damoiseaux & Greicius, 2009). Although this is true to a large extent, structure and function relationship is not straightforward. For example, although the quality of structural connections can have a direct influence on functional connectivity (Damoiseaux et al., 2006; Greicius et al., 2009; Hagmann et al., 2008; Huang & Ding, 2016), it has also been shown that functional connectivity can persist—despite dramatic cortical structural changes (e.g., for resting‐state connectivity, see Tyszka et al., 2011 or O'Reilly et al., 2013). Moreover, a well‐studied example of nonstraightforward structure and function relationship is the phenomenon of “blindsight” (Bridge et al., 2008). These experiments clearly showed that participants with partial loss of the primary visual cortex are able to make remarkably accurate psychophysical judgments about the stimulus that is presented in their blind visual field. Thus, possibly intact functional connectivity may serve as a compensating mechanism for structural changes in our patient (Honey et al., 2009; Yang et al., 2016).

Here, we examined structural (microstructural integrity) and functional connectivity in major white matter pathways that serve these specific perceptual abilities as well as localized activity and functional connectivity of these category‐selective areas to affected early visual areas in bilateral occipital pachygyria patient (NG 367‐1) and a matched control group. We investigated structural and functional connectivity in major white matter pathways to explore brain‐wide connectivity as well as localized neural activity and connections in face, place, and object category‐selective areas.

## 2 MATERIALS AND METHODS

### 2.1 Participants

#### 2.1.1 Case

The patient, a 37‐year‐old (at the time of the study) female, has prominent bilateral smoothing and thickening of the LOC, a structural abnormality that has been tied to a mutation in the laminin γ3 gene: *LAMC3* (Barak et al., 2011). Beyond the occipital area, Urgen et al. (2019) found significant structural changes in both gray and white matter morphometric indices throughout the brain. The patient is neurologically intact with average intelligence, has completed 12 years of schooling, and has been able to successfully perform all tasks in her private and professional life. She has been prescribed valproic acid, levetiracetam, pregabaline, and topiramate due to staring and blinking spells. Further patient details can be found in Urgen et al. (2019). The patient's perceptual abilities had been assessed by us previously in a separate study (Urgen et al., 2019) and found—with the exception of spatial attention—to be largely intact.

The study was in accordance with the declaration of Helsinki and was approved by the Research Ethics Committee of the university. The patient gave written consent prior to participating in this study and was compensated for her time of participation.

#### 2.1.2 Healthy controls

Nine age (mean age = 36.58, standard deviation = 3.68) and sex‐matched healthy participants with normal or corrected vision were included as a control group in the study. Data from one of the controls (age = 38) were also used for a white matter atlas, and one additional control (age = 33) participated in a functional localizer scan. All participants gave informed consent prior to participating in the study and were paid for their participation.

### 2.2 Data acquisition and protocols

High‐resolution, three‐dimensional Magnetization Prepared RApid Gradient Echo (MPRAGE), T1‐weighted anatomical images (TR = 2600 ms, TE = 3.02 ms, flip angle = 8, FOV = 256 × 224 mm^2^, voxel size 1 × 1 × 1 mm^3^, number of slices = 176, acceleration factor (GRAPPA) = 2) were acquired using a 3 Tesla scanner (Magnetom Trio, Siemens AG, Germany) with a 32‐channel phase‐array head coil.

For Diffusion Tensor Imaging (DTI) scans, we used a multiband (MB) diffusion‐weighted echoplanar imaging (EPI) sequence (voxel size = 1.8 × 1.8 × 1.8 mm^3^; TR = 3802 ms; TE = 86.6 ms; flip angle = 78 degrees; MB = 2; FOV = 210 × 181 mm^2^; slice orientation = axial; phase encode direction = anterior‐posterior; number of slices = 81; *b* = 0, 1000 s/mm^2^, diffusion directions = 64). MB‐EPI DTI parameters were optimized by us for whole brain coverage.

Resting‐state functional data were acquired with a MB gradient‐echo (GE) EPI sequence (voxel size = 1.8 × 1.8 × 1.8 mm^3^; TR = 1005 ms; TE = 27.4 ms; flip angle = 42 degrees; FOV = 208 × 186 mm^2^; slice orientation = parallel to calcarine sulcus; phase encode direction = anterior‐posterior; number of slices = 72). Resting‐state scans took about 10 min. Each participant completed one scanning session, starting with the structural scan, followed by MB‐EPI DTI and MB‐EPI resting state functional magnetic resonance imaging (fMRI) scans.

The patient and a matched healthy individual also participated in a functional localizer scan, intended to identify the fusiform face area (FFA, the PPA, as well as the lateral occipital complex (LOC). These areas were localized using standard procedures (Epstein et al., 1999; Grill‐Spector et al., 2001; Kanwisher & Yovel, 2006). For the face‐place localizer, blocks of unfamiliar faces and places were alternated with blocks of uniform gray background (“blank” condition). For the object localizer, blocks of objects and scrambled objects images were alternated with “blank” blocks. In a block, 20 randomly chosen images were presented for 300 ms followed by 200 ms blank, resulting in a stimulus presentation rate of 2 Hz. Each run consisted of 18 blocks (three conditions repeated six times) 8 TRs, as well as 5 TRs “blank” at the beginning and end of the run, resulting in a total of 154 TRs (TR = 2 s). Each localizer scan (faces–places–blank; objects–scrambled objects–blank) was repeated twice. Each run took about 5 min. The patient and the control participant were told to carefully look at each image as questions may be asked about the images later. We decided against a classic fixation task during the scan, because this type of task proved to be too stressful for the patient.

### 2.3 Analysis

#### 2.3.1 Tract‐based atlasing

**FIGURE 2:**
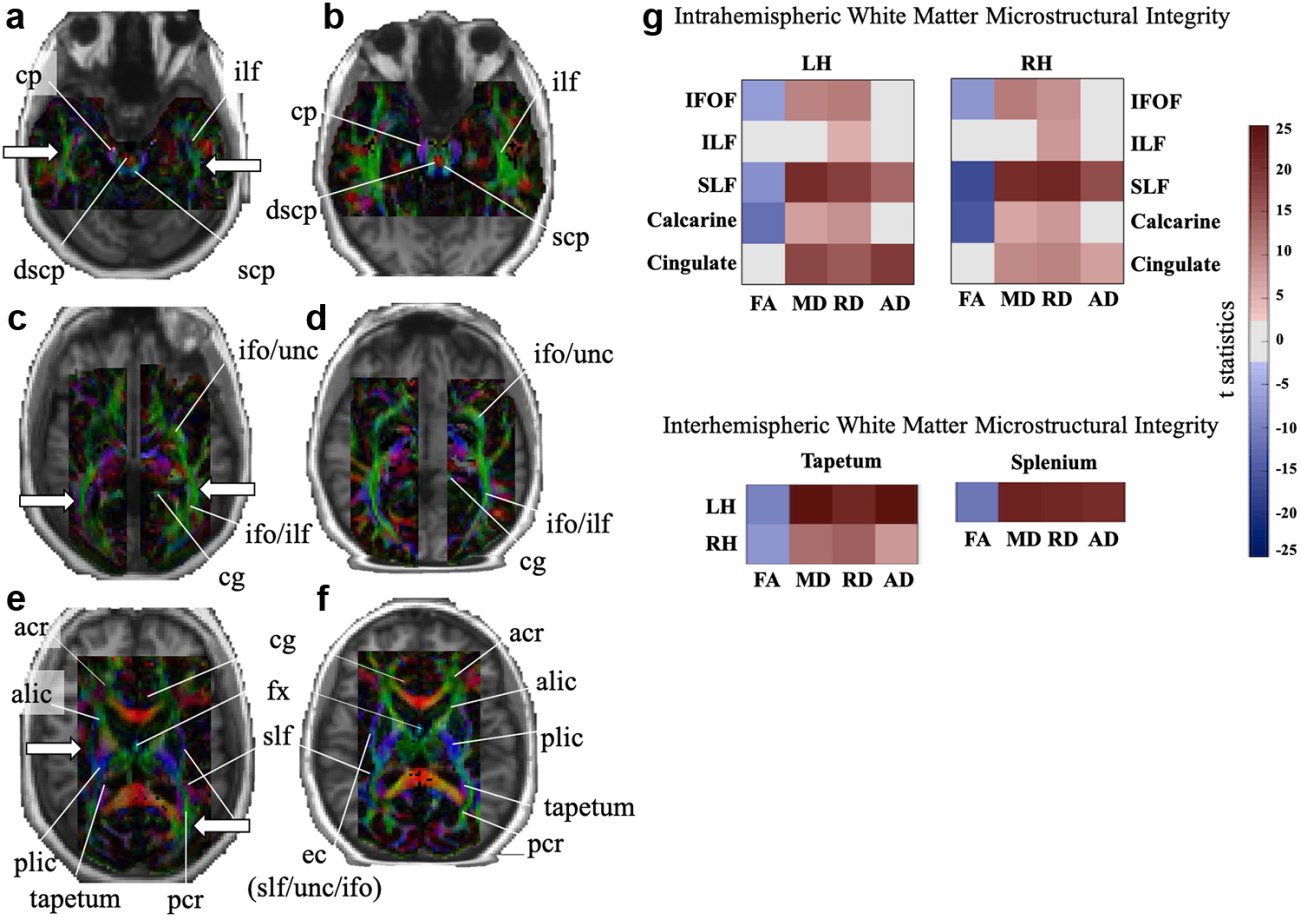
White matter tract atlas of the patient and a matched healthy control participant. This figure depicts tracts of the brain stem, projection, and association fibers. To the trained eye fiber volume appears to be reduced in bilateral IFOF, ILF, SLF, and posterior corona radiata. White arrows point to affected white matter tracts in the patient. Images are labeled color FA maps coregistered onto T1‐weighted anatomical images (following radiological convention) Label glossary: ac, anterior comissure; acr, anterior region of corona radiata; alic, anterior limb of internal capsule; cbt, corticobulbar tract; cg, cingulum; cst, corticospinal tract; dscp, decussation of superior cerebral peduncle; icp, inferior cerebral peduncle; ifo, inferior fronto‐occipital fasciculus; ilf, inferior longitudinal fasciculus; pcr, posterior region of corona radiata; plic, posterior limb of internal capsule; scr; superior region of internal capsule; sfo; superior fronto‐occipital fasciculus; slf, superior longitudinal fasciculus; unc, uncinate fasciculus. The supplementary atlas provides analogs panels for the entire brain

Diffusion‐weighted images were analyzed with FSL 5.0 (FMRIB Image Analysis Group, Oxford, UK). We first performed automatic segmentation and eddy current correction, after which FA maps were calculated using the FSL Diffusion Toolbox (FDT 2.0). Maps of the patient and one control participant were coregistered with corresponding T1‐weighted images, and major white matter tracts were labeled by hand by an experienced neuroradiologist (KKO) after careful visual inspection (also see Figure 2).

#### 2.3.2 White matter microstructural integrity

FA, mean diffusivity (MD), axial diffusivity (AD), and radial diffusivity (RD) maps were calculated using the FDT 2.0. FA is a measure of the directionality of the diffusion. Both high and low FA values have been associated with abnormal structure and function (Hoeft et al., 2007; Thomason et al., 2010), yet increases in FA usually correlate with increases in functional connectivity (van den Heuvel et al., 2008), as well as improved cognitive abilities (Penke et al., 2010). MD is a measure of overall diffusivity (Counsell et al., 2006). Its subcomponents, AD and RD, index the diffusivity in the longitudinal direction (approximately parallel to the main axon bundle) and transversal direction (perpendicular to the main axon bundle), respectively (Qiu et al., 2008). All indices (FA, MD, AD, RD) are computed from the same eigenvalues of the diffusion tensors, and their values often correlate.

FA is the most commonly used index of microstructural integrity to be associated with cognitive function, and diffusivity indices help interpret changes in FA. For example, they allow us to determine, whether decreases in FA, linked to changes in fiber density and coherence (Beaulieu, 2002) occurred due to fiber loss, interstitial water increase, gliosis, early demyelination, neuronal loss, or axonal damage (e.g., see Penke et al., 2010, or Burzynska et al., 2010). For example, elevated MD values have been associated with atrophy in tissue density or damage, whereas increases in RD might be due to demyelination, or lesser membrane density (Bonekamp et al., 2007; Dubois et al., 2008; Song et al., 2002), changes in the axonal diameters, or density. Decreases in AD is associated with axonal injury (Kim et al., 2006). Although an increase in AD could be due to more fiber coherence (Dubois et al., 2008), it could also indicate the destruction of neurofibrils or axonal degeneration (Song et al., 2003), but also see (Wheeler‐Kingshott & Cercignani, 2009) arguing for a cautious interpretation of changes in RD and AD. In general, maturating white matter is highly associated with increased anisotropy due to the structure of the axonal cell membranes (Qiu et al., 2015). We will focus our investigation of white matter microstructural integrity of the patient on the FA index, because it is the most commonly used when associating white matter structural changes with cognitive function. However, as we would like to determine the root of any potentially measured changes in FA in the patient, we will complement this index with measures of MD, RA, and AD.

#### 2.3.3 ROI definition and fiber tracking

We wanted to investigate the microstructural integrity along major white matter pathways between the occipital and temporal region and other parts of the brain as well as the integrity of interhemispheric connections, especially those known to influence the hierarchical processing in higher level vision (Furl, 2015). Thus, regions‐of‐interests (ROIs) along the inferior longitudinal fasciculus (ILF), superior longitudinal fasciculus (SLF), and inferior fronto‐occipital fasciculus (IFOF) tracts were defined by author KKO who is an experienced neuroradiologist. All ROIs are shown in Supporting Information Figure 1. As these pathways contain both long fibers as well as short connections, we placed ROIs also as waypoint seeds in the frontal, optic radiation, and occipital parts of the IFOF, and the anterior, superior, inferior, temporal, and posterior parts of the ILF. In addition, ROIs on the bilateral calcarine sulcus, cingulate cortex, SLF, splenium, and tapetum were created to investigate structural integrity in tracts that emanate from the cortical areas close to the pachygyric occipital regions. Figure S1 in the Supporting Information shows all ROI locations and Table S1 provides corresponding Montreal Neurological Institute (MNI) coordinates.

Using FSL's Probabilistic Tracking Tool, FDT version 5.0., we registered each participant's data to the MNI 2 mm standard space and then performed seed‐based tractography, eliminating fibers that do not pass through our ROIs. In all probabilistic tract calculations (except for the splenium), a binary midline mask was used as an exclusion mask to restrict computations to the target hemisphere (Figure S2). This restriction was essential for interhemispheric comparisons. We computed FA, MD, RD, and AD values for each tract and compared values between patient and healthy controls using nonparametric bootstrapping (see below).

#### 2.3.4 Resting‐state analysis

Preprocessing of the functional data was performed with the Statistical Parametric Mapping software (SPM8, http://www.fil.ion.ucl.ac.uk/spm/software/spm8/) implemented in MATLAB (Mathworks Inc., SherbornMA, USA). Preprocessing in SPM8 included image realignment, slice acquisition‐time correction, functional, and anatomical images coregistration. Functional images were normalized to the MNI standard by fitting mean functional images to a single reference EPI standard SPM template and were smoothed with a 6 mm Gaussian kernel to reduce spatial noise.

Next, for each participant, we created spherical gray matter ROIs that were directly adjacent to the white matter ROIs listed in Table S1. Each ROI was smoothed with a 6‐mm‐radius Gaussian kernel. FMRI time series were extracted and coherence scores were calculated between pairs of ROIs for each participant and the patient using the Welch's periodogram method (with a 64 time point discrete Fourier transform, Hanning window—to reduce leakage—and a 32‐point overlap). Coherence, Coh*_xy_*(*λ*), between two time series, *x* and *y* at frequency *λ* was measured by using the equation:

(1) $$Coh_{xy}\left(\lambda \right)={\left|R_{xy}\left(\lambda \right)\right|}^{2}={\left|f_{xy}\left(\lambda \right)\right|}^{2}f_{xx}\left(\lambda \right)f_{yy}\left(\lambda \right),$$

where *R_xy_* (*λ*) is the complex‐valued coherency of *x* and *y*, *f_xy_* (*λ*) is the cross‐spectrum of *x* and *y*, and *f_xx_* (*λ)* is the power spectrum of *x* (Brillinger, 2001). The coherence method is thought to be a more reliable method than simple correlational comparisons because it is not affected by differences in the hemodynamic response profile between brain regions (Sun et al., 2004). The hemodynamic response may act as a bandpass filter (typically in the range of 0–0.15 Hz) on neural activity (Aguirre et al., 1997); therefore, we performed each coherence calculation on the mean frequency scores within this range. All calculations were performed in Python using the SciPy library (https://docs.scipy.org/). Coherence values were compared between the patient and control group with a one‐sample *t*‐test as well as nonparametric bootstrapping (see below).

#### 2.3.5 Functional localization

Preprocessing and general linear model (GLM) analyses were performed with FEAT (FSL 5.0, FMRIB Image Analysis Group, Oxford, UK). Preprocessing steps include image realignment, slice acquisition‐time correction, functional, and MNI 2 mm standard space coregistration. The GLM was created from two runs for each localizer (localizer 1: face vs. place, localizer 2: object vs. scrambled object). Results were projected onto the respective coregistered T1‐weighted anatomical structures.

#### 2.3.6 Comparison of functional localizers in the patient and control

The dice coefficient measures the spatial overlap between any given two spatial maps (Novosad et al., 2020). We compared the spatial overlap between the statistical maps obtained from the GLM analysis of the face, place, and object localizer task‐based fMRI data. Maps were thresholded at 4 (minimum) and 7.8 (maximum) *z*‐score for face and place localizers for both patient and control. In the object localizer comparison, statistical maps were thresholded at 4 (minimum) and 7.8 (maximum) *z*‐score for the patient; and at 2.6 (minimum) and 7.8 (maximum) *z*‐score for the control.

#### 2.3.7 Nonparametric analysis for functional and structural connectivity analyses

As scores of a single patient were compared to a group of healthy controls, classical statistical techniques cannot be applied. Thus, we opted for combining three measures to determine whether patient and control groups scores differed. (1) Using nonparametric bootstrapping (Efron, 1979) and sampling with a replacement, we determined the 99% confidence intervals (CIs) of the control group mean diffusion indices and coherence scores (computed from the resting‐state fMRI), for each ROI. (2) We conducted one sample *t*‐tests comparing patient and control group mean diffusion indices and coherence scores. To correct for false‐positive inflation at multiple comparisons, we employed the FDR procedure by Benjamini and Hochberg (1995). (3) We determine whether the patient's mean score lies outside the range of the control group raw scores (summarized in Table S2 for white matter indices and Tables S9a–f for functional connectivity). If the patient's mean score lies outside the corresponding estimated control group confidence interval, outside the range of the control group raw scores*, and* the one sample *t*‐test comparing patient score and controls is significant at the P FDR criterion of at least P FDR < 0.05, we report a given region to be significantly different between patient and controls.

#### 2.3.8 Comparison of functional and structural connectivity

Pearson's correlation coefficient (R) was calculated for functional and structural connectivity nonparametric analyses comparison. Diffusion tensor imaging scalars (FA, MD, RD, AD) along white matter tracts of interests (IFOF, ILF, SLF as well as calcarine, cingulate, and tapetum structural connections) were compared with each functional region of interest pair along the same pathways.

## 3 RESULTS

We conjectured that object and face perception in the patient NG 367‐1 could be maintained at a normal level of functioning due to increases in microstructural integrity in white matter pathways and/or due to increases in functional connectivity between cortical areas connected by these pathways. To examine the two possibilities of brain‐wide connections, we compared microstructural integrity and functional connectivity in intra‐ and interhemispheric major visual structural pathways between the patient and a matched control group, as well as functional connectivity between face, place, and object category‐selective areas and early visual cortex.

### 3.1 Intra‐ and interhemispheric microstructural integrity

Figure 2 depicts color‐coded FA values along major white matter pathways in the patient (Figures 2A, C, and E) and a matched healthy participant (Figures 2B, D, and F). In Figure 2, panels from A to F indicate axial slices from inferior to superior, identifying brain stem (A and B), projection (A and B), and association fibers (A–F) in both patient and a healthy individual. At a first glance, there seems to be an overall reduction of white matter fibers in several intrahemispheric white matter tracts such as IFOF, ILF, SLF, or the posterior corona radiata in the patient, but also an apparent increase in fiber volume in the anterior corona radiata (Figures 2A, C, and E). We also measured intrahemispheric and interhemispheric microstructural integrity along major structural white matter pathways that are known to play a role in visual function, including face and object perception. Overall, with the exception of the ILF (intrahemispheric) and cingulate (interhemispheric), microstructural integrity of the patient, as indicated by decreases in FA values in Figures 2G (Tables 1 and 2 list corresponding numerical values and results of all statistical tests for intrahemispheric microstructural integrity and by Tables 1 and 2, which show the corresponding numerical values (as well as results of all statistical tests for intra‐ and interhemispheric microstructural integrity, respectively), appeared to be compromised.

**TABLE 1:** White matter diffusion indices. Highlighted in bold are the significant differences, that is, the *t*‐test is significant and the patient's value is outside the 99% confidence interval, and outside the range of the raw scores of the control group (*p < .05, **p < .01, ***p < .001, all FDR corrected, and ^ inside the range of control group [Table S2]). See Figure 2 for a corresponding graphical view of the statistical test results

Tracts		Control Group 99% CI	Patient	*t* test (df = 8)	*p* values
IFOF	FA	**L:0.3319−0.4338** **R:0.3276−0.4107**	**0.223** **0.228**	**−6.7399** **−7.4275**	<.0001*** .0001***
	MD(×10^3^)	**L:0.7812−0.8668** **R:0.8078−0.8540**	**1.029** **0.951**	**10.7715** **11.5250**	<.0001*** <.0001***
	RD(×10^3^)	**L:0.5908−0.7011** **R:0.6238−0.7063**	**0.929** **0.837**	**11.1335** **9.2239**	<.0001*** <.0001***
	AD(×10^3^)	L:1.13078−1.2568 R:1.1259−1.1906	1.233 1.180	2.0515 0.8884	.0743 .4002
ILF	FA	L:0.3025−0.3385 R:0.2845−0.3379	0.3048 0.2411	−1.7141 −5.9750	.3048 <.0001^
	MD(×10^3^)	L:0.8030−0.8442 R:0.8033−0.8436	0.872 0.918	5.3366 10.3327	.0007^ <.0001^
	RD(×10^3^)	**L:0.6593−0.6908** **R:0.6563−0.7109**	**0.717** **0.792**	**5.7877** **8.5232**	.0004** <.0001***
	AD(×10^3^)	L:1.0793−1.18444 R:1.0733−1.14778	1.185 1.164	3.9356 4.9557	.0043^ .0011^
SLF	FA	**L:0.2730−0.3133** **R:0.3053−0.3249**	**0.216** **0.237**	**8.5442** **−16.9594**	<.0001*** <.0001***
	MD(×10^3^)	**L:0.8397−0.8812** **R:0.8498−0.9167**	**1.062** **1.199**	**20.8271** **20.5553**	<0.0001*** <.0001***
	RD(×10^3^)	**L:0.7098−0.7597** **R:0.7150−0.7794**	**0.949** **1.070**	**18.5844** **21.5479**	<.0001*** < 0.0001***
	AD(×10^3^)	**L:1.0986−1.2049** **R:1.0741−1.1481**	**1.277** **1.450**	**13.8128** **16.8246**	<.0001*** <.0001***
Calcarine	FA	**L: 0.2911−0.3118** **R: 0.2809−0.2985**	**0.2442** **0.2286**	**−11.8691** **−15.0230**	<.0001*** <.0001***
	MD(×10^3^)	**L: 0.8079−0.8524** **R: 0.8011−0.8440**	**0.903** **0.887**	**7.3116** **6.6975**	<.0001*** <.0001***
	RD(×10^3^)	**L: 0.6791−0.7180** **R: 0.6748−0.7164**	**0.780** **0.776**	**9.0384** **8.4796**	<.0001*** <.0001***
	AD(×10^3^)	L:1.0716−1.1513 R:1.0508−1.1641	1.155 1.110	4.4022 3.0602	.0023 .0156^
Cingulate	FA	L:0.2891−0.3141 R:0.2838−0.3081	0.2965 0.2606	−1.1341 −6.2907	.2896 <.0001^
	MD(×10^3^)	**L:0.8292−0.8668** **R:0.8472−0.8958**	**0.998** **0.983**	**17.3605** **10.0094**	<.0001*** <.0001***
	RD(×10^3^)	**L:0.6953−0.7361** **R:0.7135−0.7653**	**0.86** **0.867**	**15.2595** **10.7653**	<.0001*** <.0001***
	AD(×10^3^)	**L:1.0989−1.1721** **R: 1.1199−1.1837**	**1.275** **1.212**	**18.7984** **7.7484**	<.0001*** <.0001***

Abbreviations: FA, fractional anisotropy; IFOF, inferior fronto‐occipital fasciculus; ILF, inferior longitudinal fasciculus; L, left; MD, mean diffusivity, R: right; RD: radial Diffusivity, SLF: superior longitudinal fasciculus.

**TABLE 2:** White matter diffusion indices in tapetum and splenium probabilistic tracts suggest a disruption of interhemispheric connectivity in the patient. Highlighted in bold are the significant differences, that is, the *t*‐test is significant and the patient's value is outside the 99% confidence interval, and the range of the control group's raw scores (*p < .05, **p < .01, ***p < .001, all FDR corrected). See Figure 2 for a corresponding graphical view of the statistical test results

Tracts		Control group 99% CI	Patient	*t* test (df = 8)	*p* values
Tapetum	FA	**L:0.2843−0.2987** **R:0.2817−0.3012**	**0.2591** **0.2551**	**−9.6872** **−7.8058**	<.0001*** <.0001***
	MD(×10^3^)	**L:0.8472−0.8722** **R:0.8427−0.8792**	**1.002** **0.971**	**24.9157** **13.1798**	<.0001*** <.0001***
	RD(×10^3^)	**L:0.7176−0.7464** **R:0.7152−0.7508**	**0.873** **0.852**	**21.4350** **14.6869**	<.0001*** <.0001***
	AD(×10^3^)	**L:1.1039−1.1986** **R:1.1013−1.2384**	**1.258** **1.205**	**25.8092** **8.5079**	<.0001*** <.0001***
Splenium	FA	**0.2993−0.3110**	**0.2756**	**−10.9923**	<.0001***
	MD(×10^3^)	**0.8494−0.8887**	**1.063**	**22.0751**	<.0001***
	RD(x×0^3^)	**0.7116−0.7491**	**0.918**	**21.5889**	<.0001***
	AD(×10^3^)	**1.1293−1.2327**	**1.351**	**20.8674**	<.0001***

Abbreviations: AD, axial diffusivity; FA: fractional anisotropy; L, left; MD, mean diffusivity; R, right; RD, radial diffusivity.

Of the three diffusivity indices, only AD values of the patient were similar to healthy participants for several of the investigated tracts, for example, the bilateral ILF, IFOF, and Calcarine (both intra‐ and interhemispheric connections), suggesting that axonal damage was not at the root of the decreased FA values in these pathways (Kim et al., 2006).

### 3.2 Functional connectivity

We next tested whether the observed changes in white matter structural integrity would directly affect functional connectivity between cortical areas. Thus, we created ROIs in adjacent gray matter regions along major intra‐ and interhemispheric white matter tracts (see Table S1 for the corresponding MNI coordinates of ROIs). We compared the coherence of extracted BOLD time series from these ROIs (24 ROIs for each hemisphere) between the patient and controls. 

**FIGURE 3:**
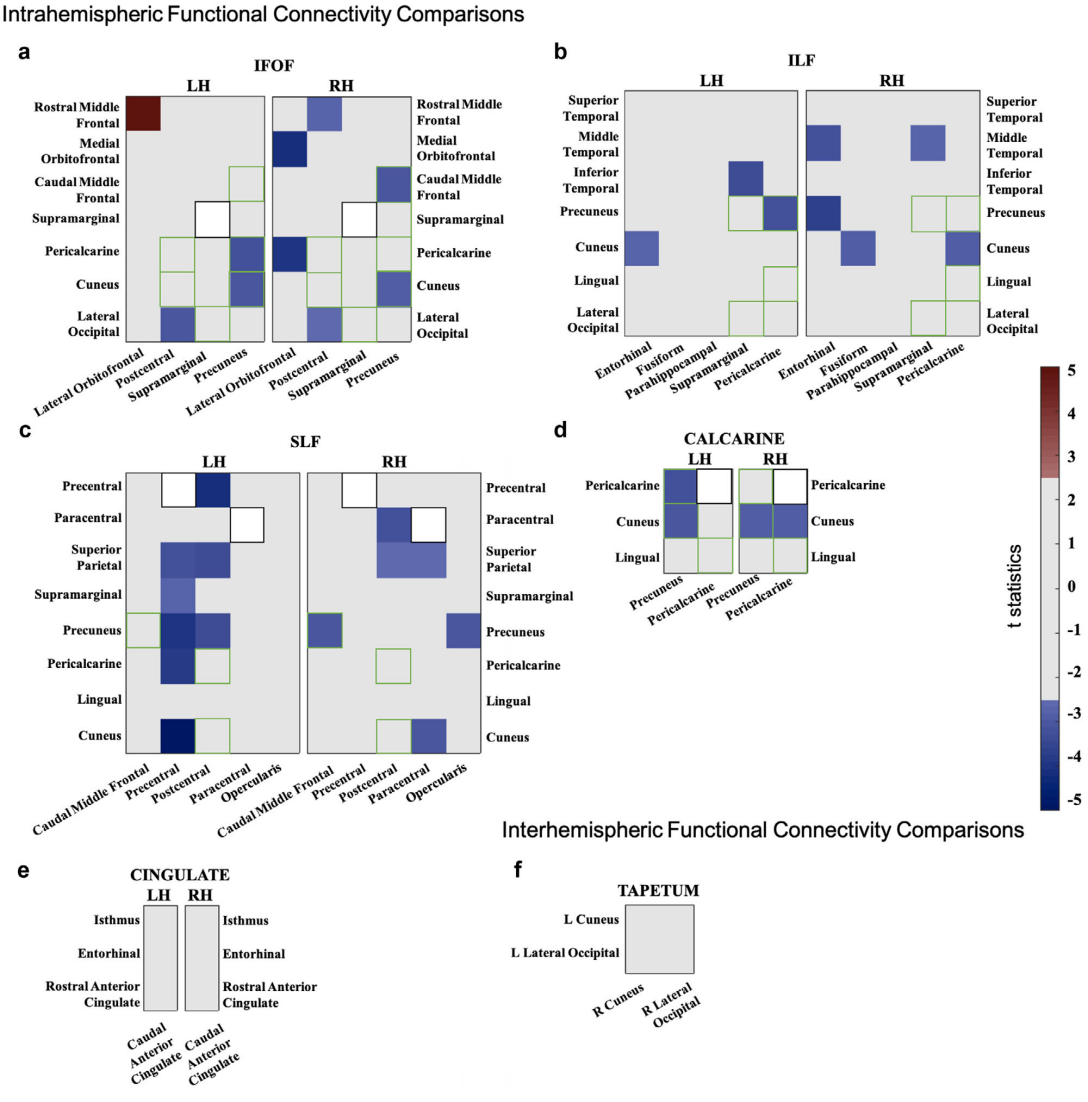
Graphical overview of intrahemispheric (A–E) and interhemipheric (F) functional connectivity differences between the patient and controls. Colors correspond to *t*‐statistics: blue indicates that the patient's connectivity values were significantly lower than those of the controls, and red indicates that the patient's connectivity values were significantly higher than those of the controls. Connectivity was computed for each hemisphere between 24 ROIs. The same ROI pairs that were examined in multiple tracts are represented with a green square, whereas the same area pairs were represented with white squares. Tables S3–S7 provide corresponding numerical values and highlight the significant differences; Table S8 shows raw coherence scores of the patient and controls for each ROI pair. All connectivity scores can be found in Figures S3 and S4 Abbreviations: AD, axial diffusivity; IFOF, inferior fronto‐occipital fasciculus; ILF, inferior longitudinal fasciculus; LH, left hemisphere; MD, mean diffusivity; RD; radial diffusivity; RH, right hemisphere; SLF, superior longitudinal fasciculus.

We report here only the ROI connections that are relevant for the white matter tracts of interests. All functional connectivity scores (24 × 24, 2304 in total) can be viewed in Figures S3 and S4 and Tables S3–S8. We followed strict criteria to determine statistically significant differences between the patient and controls including one‐sample *t*‐test, nonparametric bootstrapping analysis and raw score comparison. In Figure 3, significant functional differences between the patient and controls were represented either warm (hyperfunctional connectivity) or cold (hypofunctional connectivity) color scale for one sample *t*‐test statistics. whereas the bulk of connectivity values were similar to those of healthy controls, all but one of the significantly different functional connections indicated *reduced* functional connectivity (Figure 3). Specifically, 40% of the connections in the calcarine, 12.9% in the SLF, 16.7% in IFOF, and 11.4% in the ILF showed reduced functional connectivity in the patient, as indicated by the blue hues in Figure 3. These reductions in functional connectivity were rarely symmetric in the two hemispheres, except for the connection between the superior‐parietal gyrus and the postcentral sulcus (SLF), and between the cuneus and precuneus (IFOF and calcarine). Overall, reductions in functional connectivity predominantly occurred in connections with pre‐ and postcentral sulcus (SLF, IFOF) and precuneus (SLF, IFOF, and calcarine). A significant increase in functional connectivity was observed in the left IFOF (Figure 3, reddish hue) between the rostral middle frontal and lateral orbitofrontal gyrus. 

**FIGURE 4:**
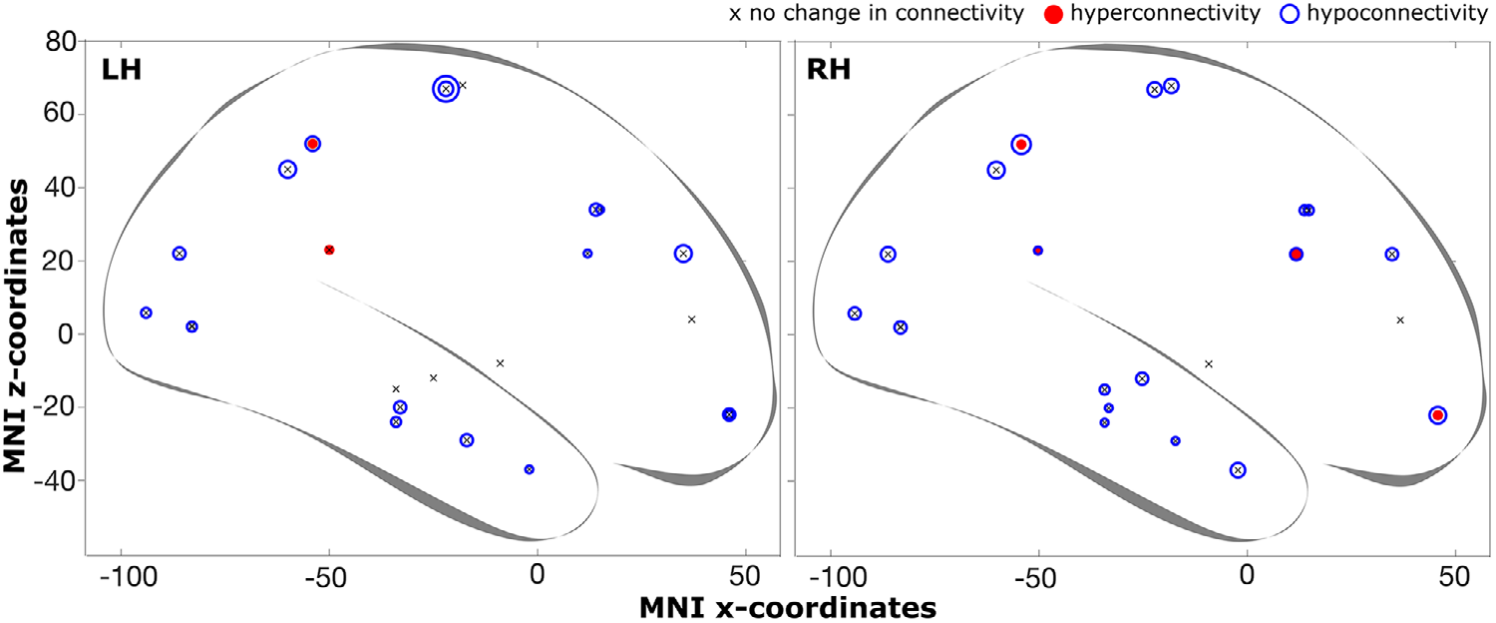
Changes in functional connectivity. This figure shows the proportion (out of 24) of changed functional connectivity for each ROI in the left and right hemispheres. The location of ROIs is indicated by their MNI coordinates (from Table S1). Red filled—and blue open circles indicate hyper and hypoconnectivity, respectively. The size of the circle indicates the relative proportion of changes in connectivity. The smallest possible value (1/24) corresponds to the smallest size symbol, for example, in the LH, at the temporal pole. Overall changes in connectivity were very low: LH Hyper: 0.007 and Hypo: 0.09 (max 9/24 for precentral); RH Hyper: 0.007 and Hypo: 0.1 (max 5/24 for precuneus)

To better assess the relative changes in functional connectivity across ROIs, we show the proportion (out of 24) of changed functional connectivity for each ROI in the left and right hemispheres in Figure 4.

By investigating long‐range structural and functional pathways in the brain, we showed brain‐wide connectivity differences in our patient. Then, we focused on localized neural activity and its functional connectivity in face, place, and object category‐selective areas.

### 3.3 Functional localization of face, place, and object category‐selective cortical areas

**FIGURE 5:**
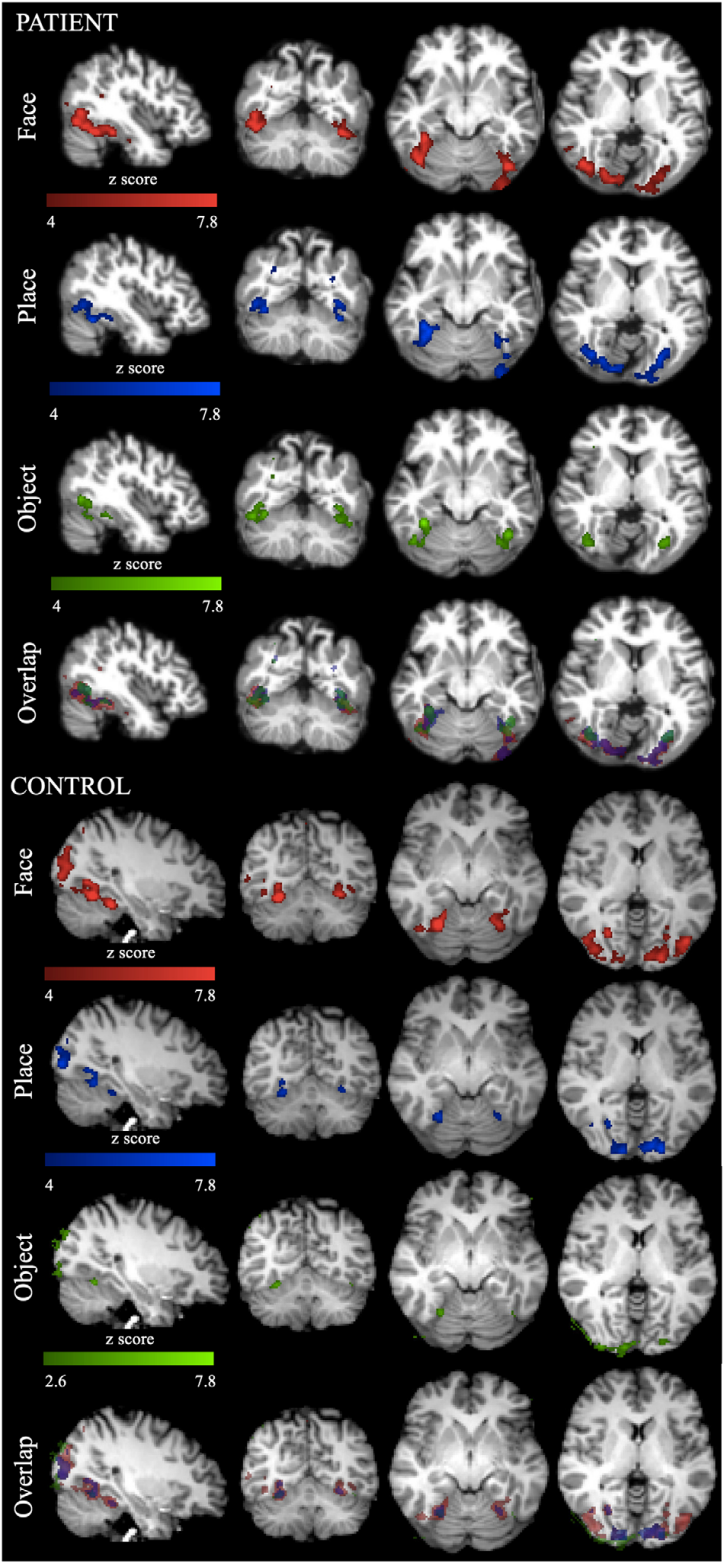
Functional localization of place, face, and object category‐selective cortical areas. Shown are sagittal, coronal, and two transversal views (from left to right) with the corresponding MNI152 2 mm standard space coordinates. Color scale represents GLM analysis results based on *z*‐statistics between 4 and 7.8 in face (red), place (blue), and object (green) localizations except object localization activity in the control participant, and *z*‐statistics were thresholded at 2.6 (minimum) in the control

Inspecting the axial view of the patient's brain in Figure 1, one suspects that several cortical visual areas should map to different locations than in the healthy brain. For example, the LOC, which is a key area involved in object perception, would be located in the midst of the largest anatomical abnormalities. The patient's cortex in that vicinity is very thick and lacks gyration, and all quantitative morphometric (Urgen et al., 2019) and microstructural integrity measures point to gross abnormalities in occipital lateral regions. At the same time, results from our present study show that microstructural integrity in more ventral regions seems to be preserved. In order to test whether it is possible to establish a qualitative mapping between the cortical activity during visual object and face recognition and white matter structural integrity, we conducted functional MRI scans. Figure 5 shows the results of the functional localization of PPA, FFA, and LOC. Interestingly, regions of activation in the patient's brain were localized in, what appears to be, an additional “inner” cortical surface, located beneath the thick pachygyric cortical surface in the occipital lobes. Moreover, activation patterns in the patient were more inferior and also showed slightly greater overlap than in the control participant. This activation pattern might also be consistent with an activation of only the ventral regions involved in object perception (Schwarzlose et al., 2008). Although the ventral regions in our patient showed less pronounced structural abnormalities, voxel‐based morphometry revealed some structural abnormalities also in these regions: For example, we found a mean curvature decrease in the right and a decrease in cortical thickness the left fusiform gyrus, as well as a significant increase in gray matter volume in right and left parahippocampus (e.g., see figure 4 in Urgen et al., 2019). However, the previous morphometric analysis might have been too coarse to have detected intact, functionally defined subregions of the fusiform gyrus (e.g., the posterior parts). Interestingly, according to Schwarzlose et al. (2008), lateral occipital regions involved in object perception are more sensitive to object location—which would predict that our patient might have some specific deficits in this kind of task. Reviewing the results of the previous visual behavior tests in Urgen et al. (2019) (their table 1), we see that the patient had indeed major impairments locating a dot on the line or locating a dot in and out of the figure.

To assess the overall similarity of the functional localizers’ spatial overlap, we calculated dice coefficients for face versus object localizer (patient: 8.8149e‐04; control:2.7114e‐04), face versus place localizers (patient: 0.0022; control: 0.0017), and object versus place localizers (patient: 0.9974 Control: 0.9966). We also measured functional connectivity between these well‐overlapped category‐selective areas and early visual areas in the patient and control group by using their resting‐state data for more generalized comparisons.

### 3.4 Functional connectivity between face, place, and object category‐selective areas and early visual areas

**FIGURE 6:**
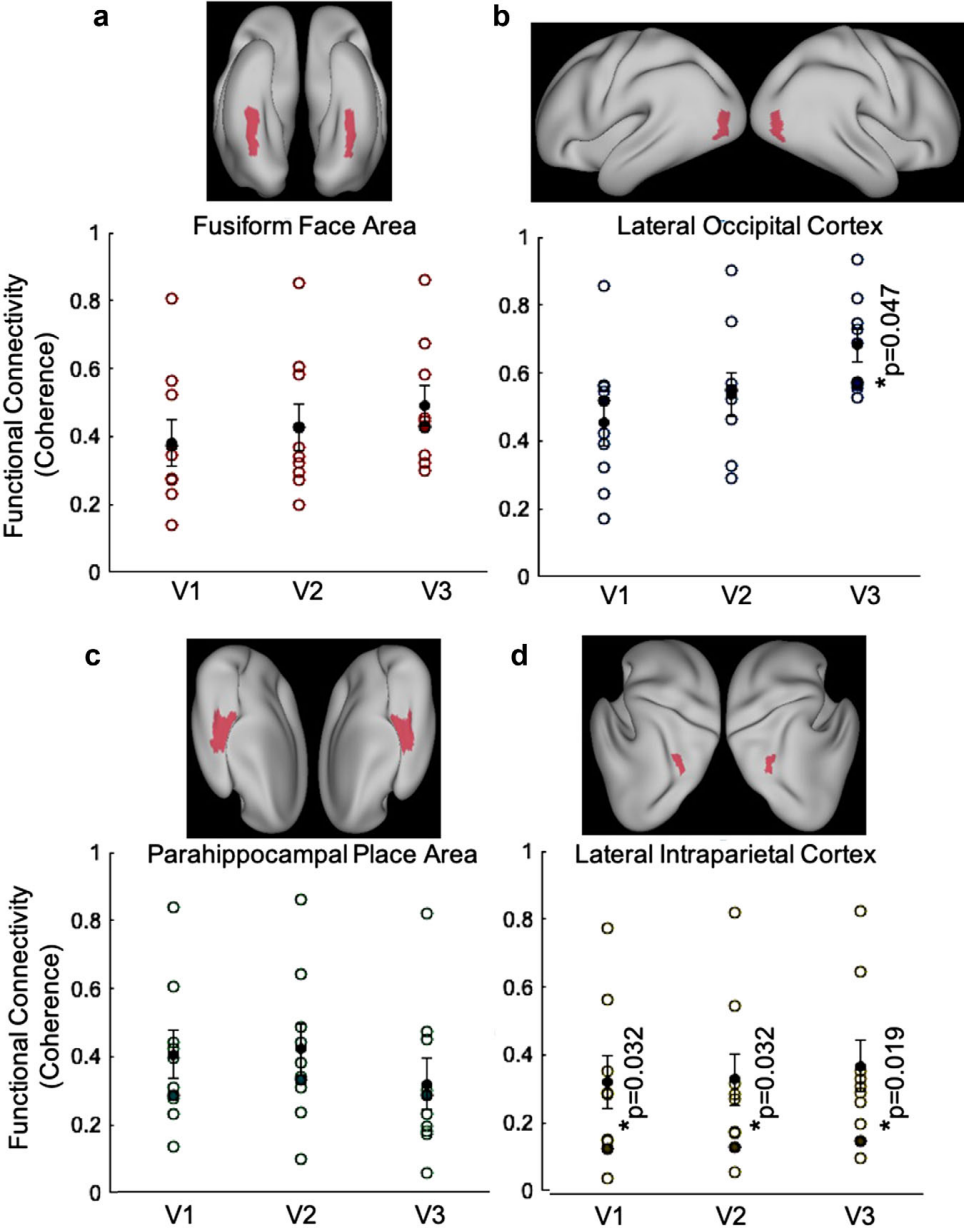
Functional connectivity between place, face, and object category‐selective areas and early visual areas (V1–V3). Functional connectivity to the category‐selective areas (fusiform face area (a), lateral occipital cortex (b), and parahippocampal place area (c) were measured for V1–V3 and indicated similar functional connectivity between the patient and controls. The lateral intraparietal cortex (d) was included in the analysis as a control area to show disrupted functional connectivity between affected areas; specifically early visual (thick and pachygyric) and lateral intraparietal cortex (polymicrogyric) areas. Each functional connectivity (coherence) data were represented with a circle in controls and the data of the patient were represented with a filled circle. Mean functional connectivity data of controls were shown with a black filled circle with ±1 standard error bars. Significant comparisons according to one‐sample *t*‐test were indicated with asterisk and the corresponding *p* values

To measure the functional connectivity to category‐selective areas (FFA, LOC, and PPA) and early visual areas (V1–V3) in the patient and control group, we computed coherence scored from the respective resting‐state data. A statistical comparison of the coherence scores (Figure 6) with a one sample *t*‐tests indicates that functional connectivity among early visual areas and category‐selective areas was not statistically different between the patient and the control group, except for the marginally significant difference for the V3‐LOC coherence (*t*(*df* = 8) = 2.346, *p* = .047), which was slightly higher in the patient. These results appear consistent with the patient's intact perceptual abilities in the face, object, and place recognition. We also included the lateral intraparietal cortex as a control area as it is reported in Barak et al. (2011).

**FIGURE 7:**
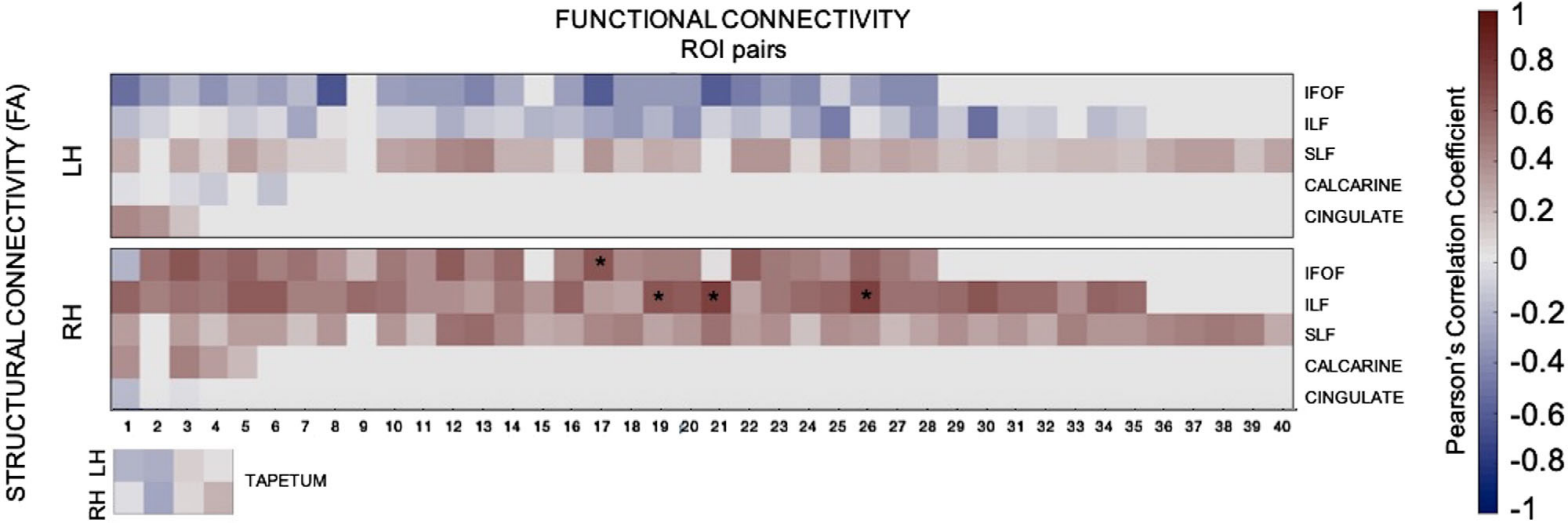
Comparison of functional and structural connectivity between the patient and controls. Pearson's correlation coefficient was calculated between fractional anisotropy values of white matter tracts of interests and functional connectivity of both intra‐ and interhemipheric region‐of‐interest pairs. Warm colors represent positive correlation coefficients, whereas cold colors represent negative correlation coefficients. Statistical comparisons that correspond to *p* < .05 were indicated with asterisk. We found a significant positive correlation between structural and three functional connectivity of three ROI pairs (precuneus‐supramarginal gyrus; cuneus‐entorhinal cortex; lingual gyrus‐entorhinal cortex) in the right ILF. Functional and structural connectivity comparisons between all diffusion indices (FA, MD, RD, and AD) of the major white matter tracts and functional connectivity of both intra‐ and interhemispheric ROI pairs were represented in Figure S5

We next compared functional and structural connectivity directly. The results in Figure 7 indicate a positive correlation between microstructural integrity along ILF and functional ROI pairs along ILF, especially in the right hemisphere. FA along ILF and functional connectivity between precuneus‐supramarginal gyrus (*r*(33) = 0.6431, *p* = .0449), cuneus‐entorhinal area (*r*(33) = 0.7226, *p* = .0183), and lingual‐entorinhal area (*r*(33) = 0.7311, *p* = .0163) in the right hemisphere was positively correlated. In addition, FA along IFOF and functional connectivity between pericalcarine sulcus and lateral orbitofrontal cortex correlation was marginally significant (*r*(28) = 0.6329, *p* = .0495).

## 4 DISCUSSION

The aim of this study was to investigate the neural mechanisms that subserve the object and face perception of a patient with a homozygous *LAMC3* gene mutation. The patient had severe bilateral smoothening and thickening of the LOC (Barak et al., 2011 ) and further structural changes in gray matter, for example, in the IT lobe (Urgen et al., 2019). Both LOC and IT are cortical regions that are critically involved in face and object perception (Epstein et al., 1999; Grill‐Spector et al., 2001; Kanwisher & Yovel, 2006). To understand how exactly this patient's brain accomplishes normal function despite these congenital structural changes, we investigated the microstructural integrity of major structural pathways involved in visual processes and functional connectivity along these pathways and compared results to a control group. Further, we investigated neural activity in face, place, and object category‐selective cortical areas and their functional connectivity with early visual areas (V1–V3). We found decreases in microstructural white matter integrity in all investigated pathways—intra‐ and interhemispheric—with the exception of the ILF (Figure 2). Significantly decreased FA values in the patient's calcarine, IFOF, SLF, tapetum, and splenium suggest a diminished fiber density and/or coherence in these pathways. These decreases were accompanied by increases in RD, which is usually associated with a compromised myelin structure (Kim et al., 2006). This points to the possibility that the *LAMC3* gene mutation directly affects the white matter integrity in these specific regions. Yet, not all pathways were affected equally. For example, ILF and cingulate also showed significant increases in RD, yet their FA values were not affected, which suggests that these pathways had only mild microstructural changes (Table 1 and 2). Conversely, SLF, tapetum, and splenium all showed significant increases in all three diffusivity indices, a pattern known from chronic extensive white matter damage (Concha et al., 2006), which suggests much more severe effects of the LAMC3 mutation on microstructural white matter integrity in these pathways.

In addition, the majority of the functional connectivity comparisons in white matter tracts of interests showed similar results to those in the control group. However, we found reduced functional connectivity between some region‐of‐interest pairs in intrahemipheric connections (Figure 3). Neural activity in the face, place, and object processing areas during the task‐based fMRI experiment for functional localizers showed similar activity in both patient and the representative control (Figure 5). Their functional connectivity to early visual processing areas (V1–V3) showed similar connectivity in the patient and the control group (Figure 6). Lastly, structural‐functional connectivity comparison revealed that both structural and functional connectivity results are positively correlated along ILF (Figure 7). In general, our functional connectivity results were not discriminative; instead, the microstructural integrity results appear to connect well to the specific perceptual abilities and deficits of the patient (Urgen et al., 2019).

### 4.1 By what mechanism are object and face perception spared?

#### 4.1.1 ILF

Our data suggest that the intact microstructural integrity in the ILF best predicted the patient's intact object and face perception abilities. This pathway projects along the temporal lobe with fibers terminating in the occipital cortex, and is thought to subserve hierarchical processing in the ventral stream, which plays a role in object recognition (Catani et al., 2003; Furl, 2015). Specifically, it is involved in mediating the fast transfer of visual signals to anterior temporal areas and neuromodulatory reverse projections from the amygdala to early visual areas that are critical for connecting visual areas to the amygdala and hippocampus (Catani et al., 2003). This pathway is also directly involved in face recognition (Fox et al., 2008; Postans et al., 2014; Tavor et al., 2014), scene perception (Gomez et al., 2015; Hodgetts et al., 2015), and object recognition (Catani & Ffytche, 2005; Catani et al., 2003; Ffytche, 2008; Fox et al., 2008; Ortibus et al., 2012). For example, lower FA values in the ILF have been associated with age‐related decline in face discrimination performance (Thomas et al., 2009). Also, an inverse relationship between FA and RD DTI metrics has been associated with cognitive function (Mayo et al., 2018; Levitt et al., 2012 ). Similarly, we showed positive correlation between structural connectivity FA values and functional connectivity and negative correlation between structural connectivity RD values and functional connectivity along ILF (Figure 7 and Figure S5). These results indicate an intact processing pathway along ILF. Consistent with the results of white matter microstructural integrity, functional connectivity between precuneus and supramarginal gyrus, cuneus and entorhinal cortex, lingual gyrus, and entorhinal cortex that were connected by ILF pathway were mostly intact. In particular, between those areas, that are directly involved in face, place, and/or object recognition, for example, the fusiform gyrus, the IT cortex, and LOC are connected through ILF.

However, we found also some decreases in functional connectivity, for example, for entorhinal cortex (LH and RH), supramarginal gyrus (LH and RH), pericalcarine (LH and RH), and even fusiform area (RH). The entorhinal cortex plays a role in visual memory (impaired in patient) and integrating spatial information into object representations (Yeung et al., 2018). The entorhinal cortex is also involved in attentional modulations (Oswald et al., 2001). In addition, supramarginal gyrus plays a role in spatial working memory (Silk et al, 2010), whereas precuneus is involved in visuospatial imagery (Cavanna & Trimble, 2006) and cuneus functionally handles basic visual processing such as spatial frequency, orientation, motion, direction, and speed (Grill‐Spector & Malach, 2004). Functions of these areas are mainly consistent with impaired perceptual abilities of the patient. Our decreased functional connectivity results provided complimentary information to their impaired behavior and significantly different white and gray matter volume relationship.

#### 4.1.2 IFOF

Also, the IFOF, which connects occipitotemporal areas with ventromedial frontal cortex, has been not only associated with face recognition (Philippi et al., 2009; Thomas et al., 2008), visual recognition and conceptualization (Sarubbo et al., 2013), and visual attention (Catani & Thiebaut de Schotten, 2008; Corbetta & Shulman, 1998), but also plays a role in other function such as determining the appropriate course of action (Elliott et al., 2000) or reinforcement learning (Butter, 1969; Jones & Mishkin, 1972; Rolls et al., 1994). Although the IFOF in the patient showed an overall significant decrease in microstructural integrity, functional connectivity was reduced only slightly more than for the ILF (16.6%). However, it appeared that the reduction occurred primarily for those cortical regions that are known to play a role in visual attention, for example, precuneus, postcentral sulcus (Corbetta & Shulman, 1998), both also connected to other cortical regions via the SLF, or caudal middle frontal gyrus (Japee et al., 2015). Moreover, there was an increase in connectivity in the left lateral orbitofrontal cortex. This is a region known to play a role in several functions including decision‐making (Nogueira et al., 2017), learning by association, emotional regulation, or face identification (Rolls, 2004).

Overall, it appears that the visual perceptual abilities (and deficits) of the patient can be best accounted for by the results of the white matter structural integrity: the more ventral a pathway is located, the more likely it appears to retain its structural integrity. This would also be consistent with the activation pattern seen in the functional scans, which show predominantly ventral parts responding to object/face/place localizers in the patient. Category selective regions come in pairs, with corresponding regions in lateral (occipital) and ventral (temporal) parts of the brain (Schwarzlose et al., 2008). It could be that the intact object, face recognition behavior of the patient (Urgen et al., 2019), is subserved by a largely intact ventral visual pathway. Specific problems of the patient in localizing objects—a perceptual ability primarily subserved by dorsal pathway—would also support this view. 

### 4.2 Evidence for compromised visuospatial attention

The microstructural findings of this study are en par with the morphometry results of Urgen et al. (2019) in that both point to the patient's problems in visuospatial attention. In particular, the SLF, which connects the superior parietal lobule/intraparietal sulcus with the superior frontal gyrus/frontal eye field, has been primarily associated with spatial attention (Hoeft et al., 2007; White et al., 2008), and top‐down modulation of visual processes (Catani et al., 2002) via dorsal frontoparietal (Corbetta & Shulman, 2002; Corbetta et al., 2008; Hopfinger et al., 2000) and ventral attention networks (Hattori et al., 2018). Not only FA, but also all diffusivity indices differed significantly in the SLF between patient and controls, indicating a severely compromised microstructural integrity in this pathway.

Surprisingly, this tract's decrease in functional connectivity was only 12.9% of the total number of connections, and thus comparable to connectivity decreases in the ILF (11.4%). Therefore, functional connectivity seemed much more intact than white matter structural integrity—or the patient's visual attention deficits—might have one lead to suspect. Inspecting the functional connections that were decreased, we do find that the areas involved are known to play important roles in spatial attention, such as pre‐ and postcentral sulcus and superior parietal gyrus (nodes in the dorsal attention network, Corbetta & Shulman, 1998; Corbetta & Shulman, 2002; Grosbras & Paus, 2006), pars opercularis, caudal middle frontal gyrus, precuneus, supramarginal gyrus (nodes in the ventral network, Corbetta & Shulman, 2002), or paracentral lobule (orienting, Xiao et al., 2016).

We also found decreased structural integrity in the corpus callosum (FA, as well as all diffusivity indices). This structure is the largest fiber bundle that mediates interhemispheric communication among cortical areas and has an important role in balancing higher‐level cognitive functioning (Hofer & Frahm, 2006). Moreover, it is also involved in regulating the mutual inhibition between bihemispheric posterior parietal areas in visuospatial attention (Plow et al., 2014). This potentially impaired balance mechanism between the bilateral SLF pathways would be consistent with the patient's visuospatial attention deficits. 

### 4.3 Agreement between structural and functional indices

Urgen et al. (2019) found marked gray matter morphometry changes in occipital and temporal cortices in the patient's brain. They also found increases in WMV in the patient's IT and right lateral occipital cortices and postulated that this might be a mechanism to compensate for the adverse effects of gray matter changes. Specifically, they expected that this increase would reflect increases in microstructural integrity and/ or functional connectivity in pathways connecting this area. Our results do not support this idea of a compensatory mechanism, because WMV increases were also associated with FA decreases (IFOF, calcarine, or tapetum) or no change of FA values (ILF). Furthermore, decreases in WMV (as in the SLF) co‐occurred with decreases in microstructural integrity. Consequently, no consistent pattern between white matter morphometric (volume) and white matter structural indices emerged.

**FIGURE 8:**
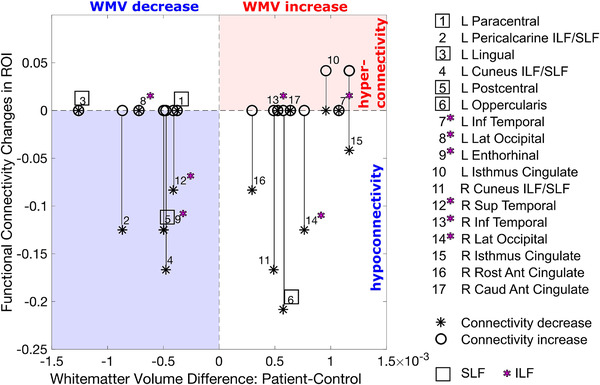
Changes in white matter volume and functional connectivity. Plotted are the proportion of significant connectivity changes for each ROI as a function of corresponding significant differences in white matter volume between patient and control group. The proportion of hypoconnectivities is multiplied by −1 before plotting. Black vertical lines connect the proportions of hypo‐ and hyperconnections for a given ROI. ROI numbers enclosed by a square are unique to the superior longitudinal fasciculus. ROI numbers with a magenta star are unique to the inferior longitudinal fasciculus. White matter volume data are obtained from Urgen et al. (2019). This plot illustrates that there was not a clear relationship between white matter volume changes and changes in functional connectivity. White matter volume in and decreases occurred in both SLF and ILF

Importantly, we do find that the white matter microstructural integrity in the ILF was preserved. Also, the functional connectivity in the ILF *was* largely intact, however, that was not unique to this particular pathway—all other investigated white matter tracts showed a similarly intact functional connectivity. In the introduction, we speculated that WMV increases might point to some compensatory mechanisms, like hyper connectivity. Plotting the relationship between WMV change for ROIs common to both studies in Figure 8, we find that there is no clear relationship between these two indices, as we find decreases in functional connectivity in both ROIs with WMV increases and decreases.

Given the lack of correspondence that we see in this figure and between microstructural integrity and functional connectivity, it seems that for this patient, the properties of structural connections do not stand in direct correspondence to the degree of functional connectivity. This also suggests that functional connectivity (i.e., coherence) can persist despite cortical structural changes. Measuring the relationship between microstructural and functional integrity, we found that FA of ILF is positively correlated with multiple functional connectivity ROI pairs located within ILF pathway (Figure 7).

Taken together, it appears that only in the context of explaining the patient's deficits, for example, in visual attention, that morphometric, microstructural integrity, and functional connectivity indices yielded consistent outcomes^1^. The intact visual behavior, such as object or face perception in our patient, appears to be best accounted for by the preserved microstructural integrity of the ILF. Results of the functional scans, which show only activity in the ventral part of category‐selective cortical regions, support this view. Moreover, the patient's deficits in task involving object localization, a function primarily subserved by the dorsal visual pathway, would be consistent with this interpretation. The result, that the link between indices of cortical structure and function can at best be moderate, is in line with previous work, showing that white matter structural connectivity is not correlated to resting‐state functional connectivity (Tsang et al., 2017) or that normal resting‐state functional connectivity is possible despite missing or severed corpus callosum (Tyszka et al., 2011 or O'Reilly et al., 2013, but also see Huang & Ding, 2016 for an alternative view).

### 4.4 Gray matter or white matter—The *LAMC3* chicken and egg question

Alterations in white matter structure can have profound effects on the gray matter by affecting large ensembles of neurons (Hulshof Pol et al., 2006; Thiebaut de Schotten et al., 2005), conversely, changes in gray matter structure can influence the structure and integrity of axons (Rose et al., 2000; Wang et al., 2016). However, the specific genetic influence on human brain architecture remains poorly understood, including *LAMC3*’s direct effects on white or gray matter development. What we do know is that *LAMC3* transcription appears to peak at the interval between late development to late infancy during which the migration of postmitotic neurons to the cortical plate and the myelination of axons takes place (Barak et al., 2011). This process is one of the most prolonged developmental periods that continues to the twenties (Benes, 1989; Yakovlev & Lecours, 1967). Thus, any changes (e.g., in expression levels of *LAMC3*) during this period of development could lead to alterations in white matter structure. However, LAMC3 could also affect gray matter development directly. Laminin expression is^1^ effective in both neuronal migration and final laminar positioning of neurons in the neocortex (Kwan et al., 2012). Thus, with the current state‐of‐the‐art knowledge and tools, it is impossible to distinguish whether *LAMC3* affected white matter structure that, in turn, affected gray matter structure or vice versa.

Why alterations in the gray or white matter brought on by the homozygous *LAMC3* mutation would lead to only impairments of visual attention but not in basic visual perception, or face, and object recognition—as we found in our patient—remains an open question. It might be possible that the human brain, when faced with widespread congenital structural changes, is capable of maintaining basic and highly localized or specialized perceptual mechanisms, but cannot fully achieve higher cognitive functions, like attention, which require systematic excitation and suppression of complex networks (Silverstein et al., 2012).

### 4.5 Limitations

The biggest limitation of any single case study is to draw conclusions comparing an individual to a control group. We have here an absolutely unique individual and thus no alternative to this approach. In order to guard against false positives, we apply a very conservative criterion for statistical significance: (1) the patient's score had to lie outside the 99% bootstrapped confidence interval, (2) it had to be outside the range of the control group raw scores, and (3) FDR‐corrected *p*‐values had to be smaller than .05.

#### 4.5.1 Effects of HRF latency and shape on functional connectivity

Functional connectivity is defined by (Friston et al., 1993) as the temporal correlation of spatially remote neurophysiological events. However, this correlation method at zero lag is very sensitive to the shape of the hemodynamic response function (HRF) (Sun et al., 2004). Even in the same scan and subject, the shape of the HRF may be different in different parts of the brain (Aguirre et al., 1998), which may render correlations spuriously low. To be able to overcome this, we used coherence comparison in the frequency domain to measure how two distinct areas are coherently active during the resting state scan.

#### 4.5.2 Effects of medication

The patient's seizures are being controlled with a combination of valproic acid, levetiracetam, pregabalin, and topiramate. Although no side effects were reported by the patient or her neurologist, these drugs might nevertheless have had a potential influence on the patient's cortical structure and function. For example, it has been shown that valproic acid treatment contributes to white matter repair and neurogenesis in the ischemic brain (Liu et al., 2012). Similarly, topiramate treatment provided dose‐dependent and long‐lasting protection of developing white matter in the rodent model (Sfaello et al., 2005). Although there is no direct evidence on pregabalin effects on white matter, it has been linked to decreases in functional connectivity between components of the Default Mode Network (DMN) network (Harris et al., 2013). Even though our patient has been on medication for several years, her dosage has been much lower compared to these studies, which might have rendered the possible positive and negative effects of these mediations, or their interactions, on white matter microstructure and functional connectivity negligible.

## 5 CONCLUSION

We conclude that the neural basis of the remarkably intact object and face recognition abilities of a patient with a homozygous *LAMC3* gene mutation can be best explained by the largely intact white matter structural integrity in the pathways involved in these perceptual tasks, most notably FA in ILF. Our results indicate that the link between cortical structure and function is—more often than not—*not* a direct one. This is perhaps not entirely surprising given the complex structural and functional architecture of the brain. Our overall results provide complementary information about the effect of the LAMC3 gene on the human brain, including intact temporo‐occipital structural and functional connectivity that is compatible with preserved perception abilities.

## FUNDING

This work was funded by a grant of the Turkish National Scientific and Technological Council (TUBITAK 112K069) and a Turkish Academy of Sciences Young Scientist Award (TUBA GEBIP) and a Sofja Kovalevskaja Award by the A.v. Humboldt foundation awarded to KD. Efforts of PD is supported by Turkish National Scientific and Technological Council Bilim Insani Destekleme Daire Baskanligi (TUBITAK BIDEB) 2211 Domestic PhD Scholarship program.

## CONFLICT OF INTEREST

The authors declare that there is no conflict of interest regarding the publication of this article.

### PEER REVIEW

The peer review history for this article is available at https://publons.com/publon/10.1002/brb3.2241.

## Supporting information

SUPPORTING INFORMATION

SUPPORTING INFORMATION

## Data Availability

The data that support the findings of this study are openly available in zenodo at https://doi.org/10.5281/zenodo.4598198, reference number 4598198.

## Footnotes

1 Morphometric values, diffusion indices. and functional connectivity measures were also consistent for the entorhinal cortex, an area involved in visual memory (which was also impaired in the patient).
