# Supplementary material for: Cortical connectivity in the face of congenital structural changes—A case of homozygous LAMC3 mutation
Source: Brain Behav. 2021 Jun 14;11(8):e2241. doi: 10.1002/brb3.2241 (PMC8413815; doi:10.1002/brb3.2241)
Supplement: Supplementary file 1 — SUPPORTING INFORMATION [file BRB3-11-e2241-s001.pdf]

## SUPPORTING INFORMATION

***Probabilistic Tractography***

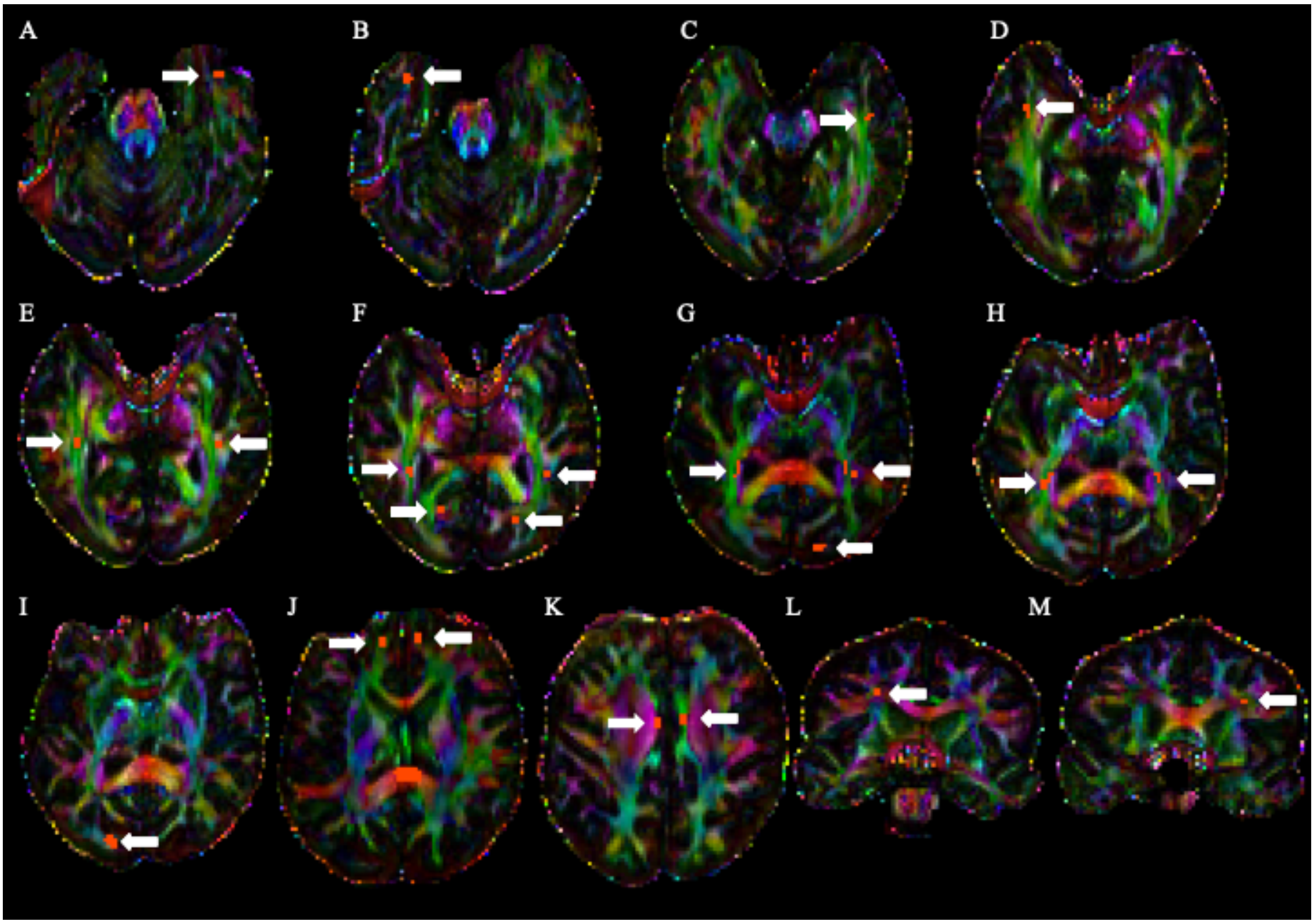


**Supplementary Figure 1. ROI locations that were used in probabilistic tractography.** A. Left anterior part of ILF, B. Right anterior part of ILF, C.Left superior lateral part of ILF, D.Right superior lateral part of ILF, E. Left and right temporal part of ILF, F. Left and right occipital part of IFOF, left and right posterior part of ILF, G. Left optic radiation part of IFOF, left and right tapetum, left calcarine, H.right optic radiation part of IFOF, left and right tapetum, I. Right calcarine, J. Splenium, left and right frontal part of IFOF, K. Left and right cingulate, L. Right SLF, M. Left SLF. White arrows show region of interests in each slice. IFOF: inferior fronto-occipital fasciculus, ILF: inferior longitudinal fasciculus, SLF: superior longitudinal fasciculus.

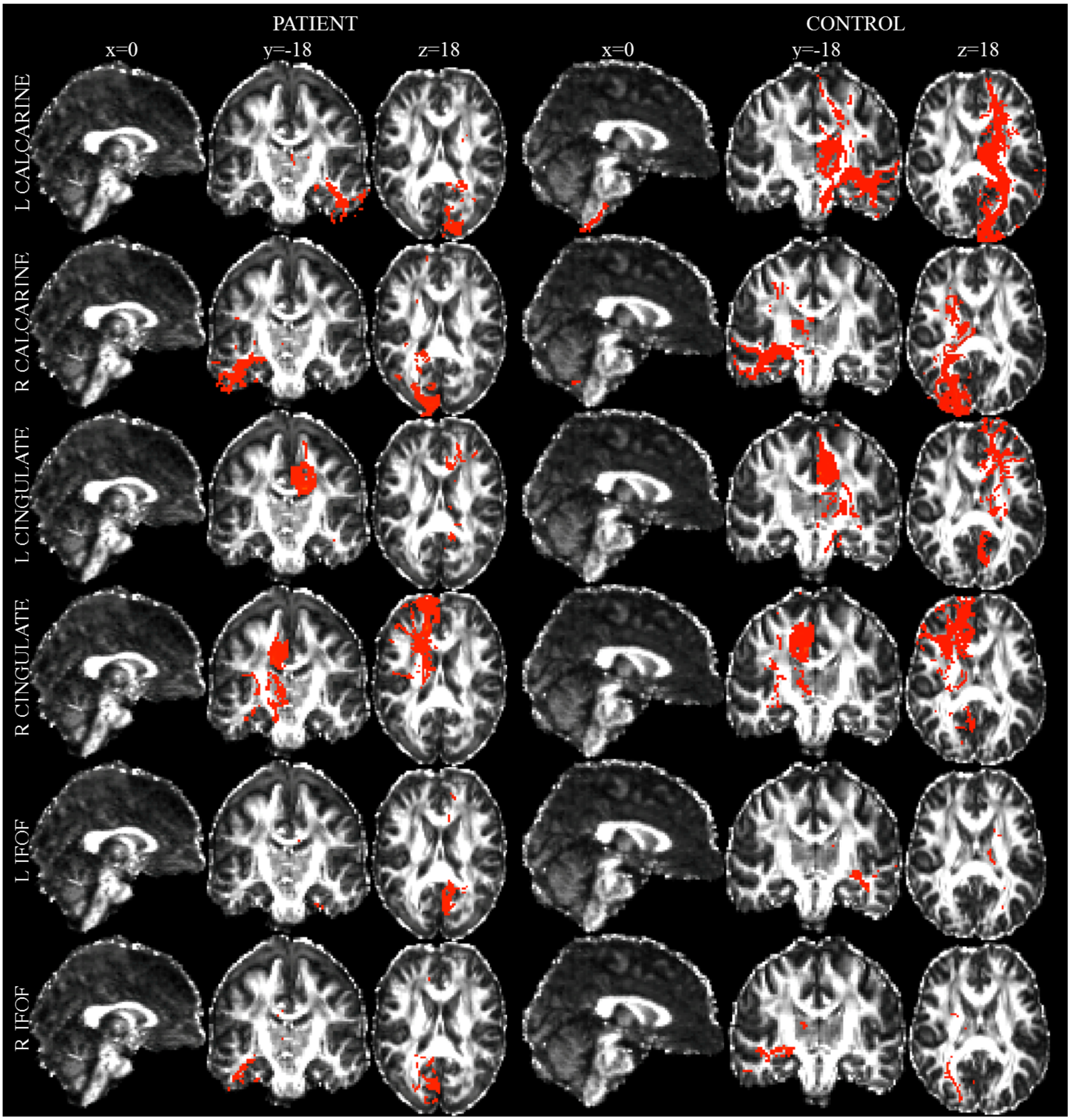


**Supplementary Figure 2. Probabilistic tractography of bilateral calcarine, cingulate, IFOF, ILF, SLF, tapetum and splenium in the patient and a representative control.**

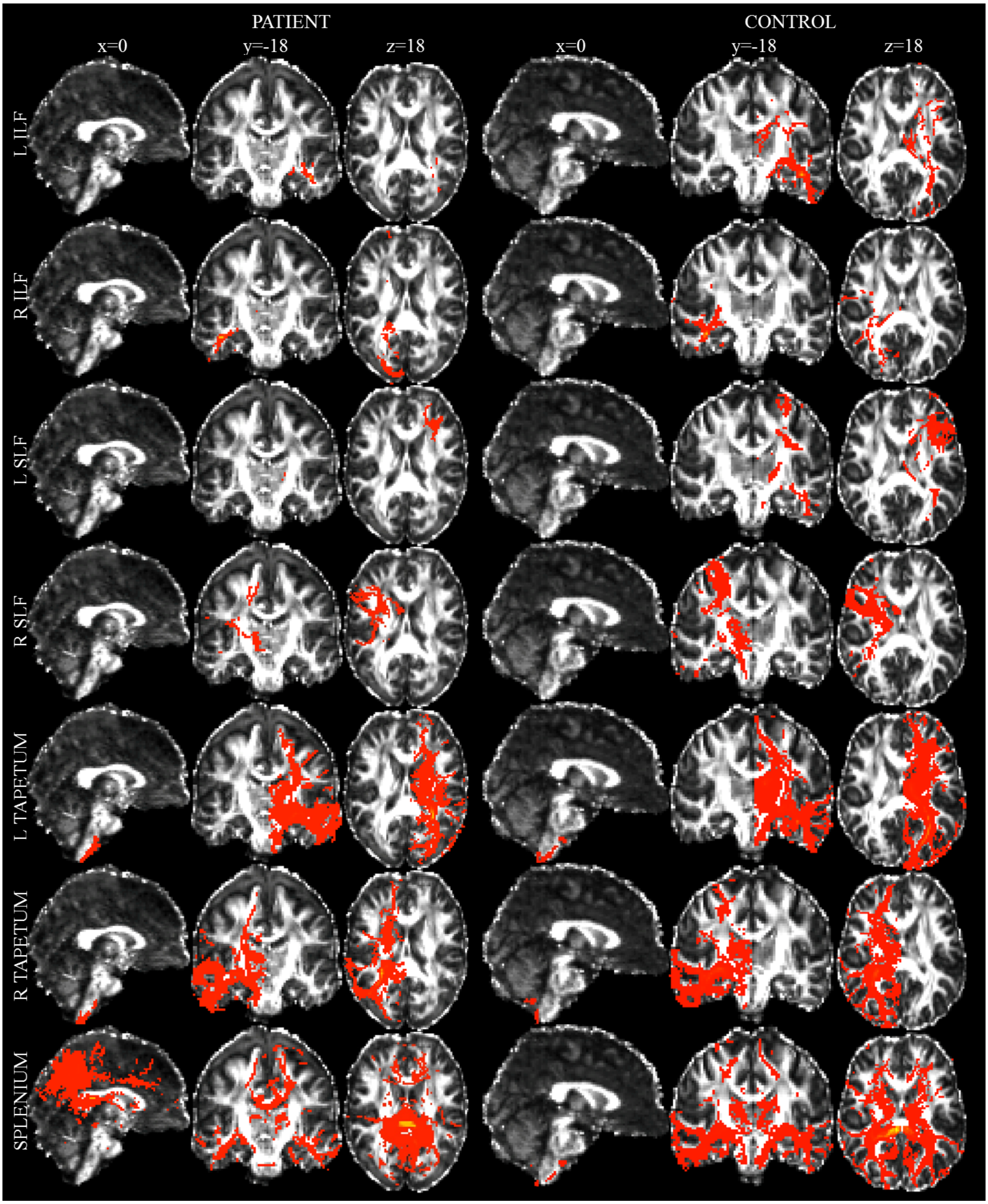


**Supplementary Figure 2. cont.**

**Supplementary Table 1. Names and MNI coordinates of ROIs that were used in microstructural integrity- and functional connectivity analysis.** Coordinates refer to the center of the spherical ROIs. G: Gyrus, S: Sulcus.

| | *Region* | *Left/right hemispheres mni coordinates (x y z)* | | | | | | |
|---|---|---|---|---|---|---|---|---|
| *IFOF* | Rostral Middle Frontal G | -38 | 35 | 22 | / | 40 | 35 | 22 |
| | Medial Orbitofrontal G | -4 | 46 | -22 | / | 6 | 46 | -22 |
| | Caudal Middle Frontal G | -38 | 14 | 34 | / | 40 | 14 | 34 |
| | Supramarginal G | -57 | -17 | -29 | / | 59 | -17 | 29 |
| | Pericalcarine S | -6 | -83 | 2.2 | / | 8 | -83 | 2 |
| | Cuneus | -6 | -86 | 22 | / | 8 | -86 | 22 |
| | Lateral Occipital G | -18 | -94 | 5.8 | / | 21 | -94 | 5 |
| | Postcentral G | -31 | -22 | 67 | / | 33 | -22 | 67 |
| | Precuneus G | -6 | -54 | 52 | / | 8 | -54 | 52 |
| *ILF* | Superior Temporal G | -54 | -9 | -8 | / | 57 | -9 | -8 |
| | Middle Temporal G | -58 | -25 | -12 | / | 60 | -25 | -12 |
| | Inferior Temporal G | -62 | -33 | -20 | / | 66 | -17 | -19 |
| | Precuneus | -6 | -54 | 52 | / | 8 | -54 | 52 |
| | Cuneus | -6 | -86 | 22 | / | 8 | -86 | 22 |
| | Lingual G | -6 | -83 | -6 | / | 8 | -83 | -6 |
| | Lateral Occipital G | -18 | -94 | 5 | / | 21 | -94 | 5.8 |
| | Entorhinal area | -24 | -2 | -37 | / | 26 | -2 | -37 |
| | Fusiform | -36 | -32 | -24 | / | 35 | -32 | -24 |
| | Parahippocampal G | -16 | -34 | -15 | / | 19 | -34 | -15 |
| | Supramarginal G | -57 | -17 | -29 | / | 59 | -17 | 29 |
| | Pericalcarine S | -6 | -83 | 2 | / | 8 | -83 | 2 |
| *SLF* | Precentral G | -31 | -22 | 67 | / | 33 | -22 | 67 |
| | Paracentral G | -16 | -18 | 68 | / | 18 | -18 | 68 |
| | Superior Parietal G | -25 | -60 | 45 | / | 27 | -60 | 45 |
| | Supramarginal G | -57 | -17 | -29 | / | 59 | -17 | 29 |
| | Precuneus | -6 | -54 | 52 | / | 8.4 | -54 | 52 |
| | Pericalcarine S | -6 | -83 | 2 | / | 8.4 | -83 | 2 |
| | Lingual G | -6 | -83 | -6 | / | 8.4 | -83 | -6 |
| | Cuneus | -6 | -86 | 22 | / | 8.4 | -86 | 22 |
| | Caudal Middle Frontal G | -38 | 14 | 34 | / | 40 | 14 | 34 |
| | Postcentral G | -31 | -22 | 67 | / | 33 | -22 | 67 |
| | Opercularis | -44 | 12 | 22 | / | 46 | 12 | 22 |
| *CALC* | Pericalcarine S | -6 | -83 | 2 | / | 8 | -83 | 2 |
| | Cuneus | -6 | -86 | 22 | / | 8 | -86 | 22 |
| | Lingual G | -6 | -83 | -6 | / | 8 | -83 | -6 |
| | Precuneus | -6 | -54 | 52 | / | 8 | -54 | 52 |
| *CING* | Isthmus Cingulate G | -6 | -50 | 23 | / | 8 | -50 | 23 |
| | Entorhinal area | -24 | -2 | -37 | / | 26 | -2 | -37 |
| | Rostral Anterior Cingulate G | -6 | 37 | 4 | / | 8 | 37 | 4 |
| | Caudal Anterior Cingulate G | -6 | 15 | 34 | / | 8 | 15 | 34 |
| *TAPETUM* | Cuneus | -6 | -86 | 22 | / | 8 | -86 | 22 |
| | Lateral Occipital G | -18 | -94 | 5.8 | / | 21 | -94 | 5 |

**Supplementary Table 2. Raw scores of white matter diffusion indices.** Shown are minimum and maximum boundaries of raw scores in healthy controls and raw scores of the patients along major white matter tracts in left (L) and right (R) hemispheres. Raw scores were provided as a complementary information of bootstrapping results that are provided in Table 1. Statistically significant results are written in bold text. ^ indicates that the patients value was inside the range of control group.

| *Tracts* | | *Min-Max Raw Scores* | *Patient's Data* |
|---|---|---|---|
| *IFOF* | FA | **L:0.2829-0.5211** | **0.2232** |
| | | **R:0.2749-0.4542** | **0.2278** |
| | MD | **L:0.8000-0.9000** | **0.9800** |
| | | **R:0.8000-0.9000** | **0.9500** |
| | RD | **L:0.5000-0.8000** | **0.9000** |
| | | **R:0.5870-0.7700** | **0.8360** |
| | AD | L:1.0930-1.2950 | 1.2330 |
| | | R:1.0740-1.2490 | 1.1800 |
| *ILF* | FA | L:0.2848-0.3683 | 0.3048 |
| | | R:0.2295-0.3645 | 0.2411^ |
| | MD | L:0.7950-0.8900 | 0.8720^ |
| | | R:0.7770-0.8710 | 0.9180^ |
| | RD | **L:0.6420-0.7110** | **0.7170** |
| | | **R:0.6000-0.7000** | **0.8000** |
| | AD | L:1.0520-1.2410 | 1.1850^ |
| | | R:1.0220-1.1650 | 1.1640^ |
| *SLF* | FA | **L:0.2323-0.3186** | **0.2164** |
| | | **R:0.2968-0.3386** | **0.2368** |
| | MD | **L:0.8000-0.9000** | **1.1000** |
| | | **R:0.0008-0.9500** | **1.2000** |
| | RD | **L:0.7000-0.8000** | **0.9000** |
| | | **R:0.7000-0.8000** | **1.1000** |
| | AD | **L:1.1000-1.2000** | **1.3000** |
| | | **R:1.1000-1.2000** | **1.5000** |
| *Calcarine* | FA | **L:0.2827-0.3272** | **0.2442** |
| | | **R:0.2712-0.3067** | **0.2286** |
| | MD | **L:0.7810-0.8830** | **0.9030** |
| | | **R:0.7840-0.8710** | **0.8870** |
| | RD | **L:0.7000-0.7000** | **0.8000** |
| | | **R:0.7000-0.7000** | **0.8000** |
| | AD | L:1.0370-1.1690 | 1.1550^ |
| | | R:1.0270-1.1420 | 1.1100 |
| *Cingulate* | FA | L:0.2657-0.3278 | 0.2442 |
| | | R:0.2724-0.3199 | 0.2606^ |
| | MD | **L:0.8000-0.9000** | **0.9800** |
| | | **R:0.8000-0.9000** | **0.9760** |
| | RD | **L:0.7000-0.8000** | **0.9000** |
| | | **R:0.7000-0.0008** | **0.0009** |
| | AD | **L:1.1000-1.1000** | **0.0013** |
| | | **R:1.0980-1.1760** | **1.2120** |
| *Tapetum* | FA | **L:0.2772-0.3109** | **0.2591** |
| | | **R:0.2743-0.3201** | **0.2551** |
| | MD | **L:0.8000-0.9000** | **0.9500** |
| | | **R:0.8000-0.9000** | **0.9600** |
| | RD | **L:0.7000-0.8000** | **0.9000** |
| | | **R:0.7000-0.8000** | **0.9000** |
| | AD | **L:1.1000-1.1000** | **1.3000** |
| | | **R:1.0710-1.1620** | **1.2050** |
| *Splenium* | FA | **L:0.2902-0.3176** | **0.2756** |
| | MD | **R:0.8000-0.9000** | **1.1000** |
| | RD | **L:0.7000-0.8000** | **0.9000** |
| | AD | **R:1.1000-1.2000** | **1.4000** |

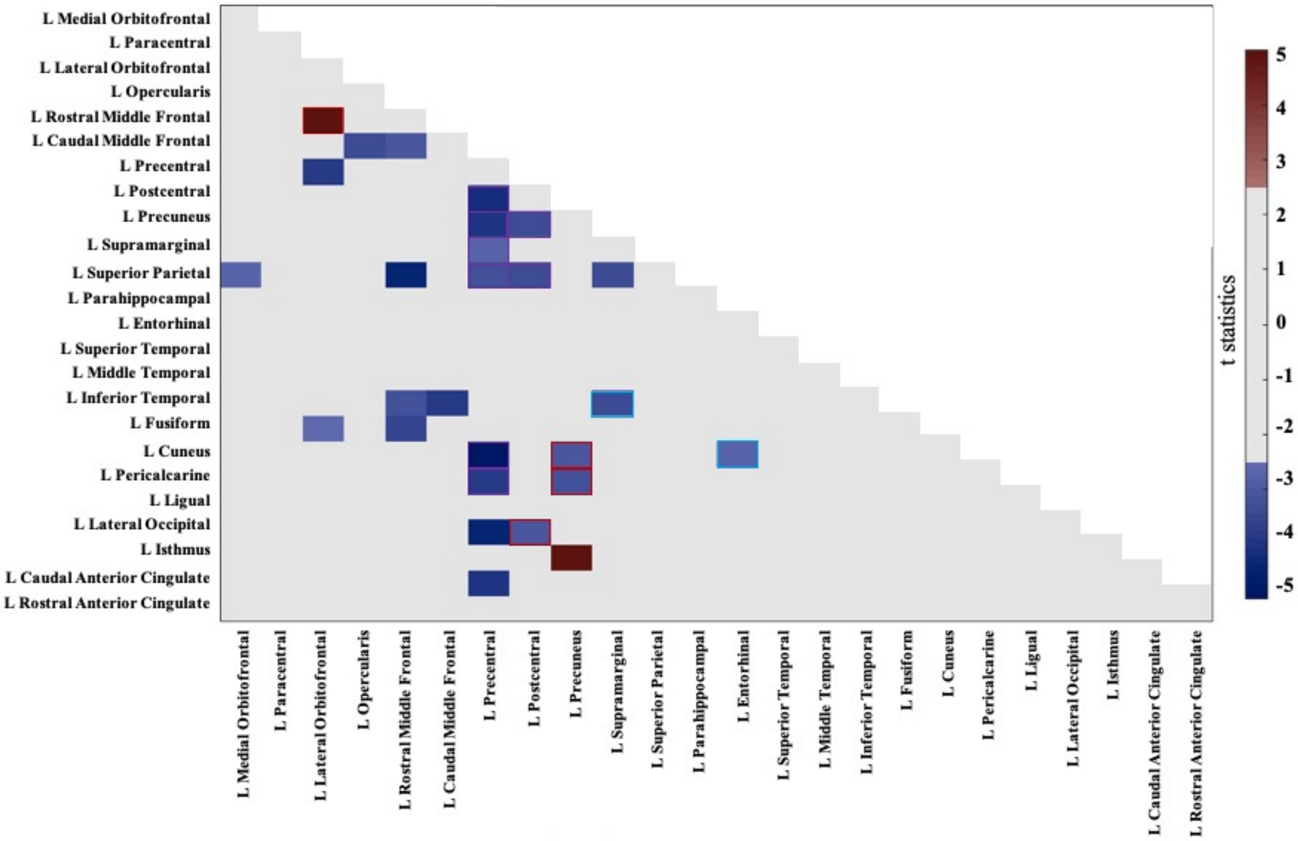


**Supplementary Figure 3. Graphical overview of coherence comparisons of ROIs in the left (L) hemisphere between the patient and controls.** Colors correspond to t statistics, blue indicates that the patient's connectivity values were significantly lower than those of the controls, red indicates that the patient's connectivity values were significantly higher than those of the controls. Non-significant differences are plotted in light gray. ROIs within inferior fronto occipital fasciculus (red), inferior longitudinal fasciculus (blue), superior longitudinal fasciculus (purple) were represented with rectangles.

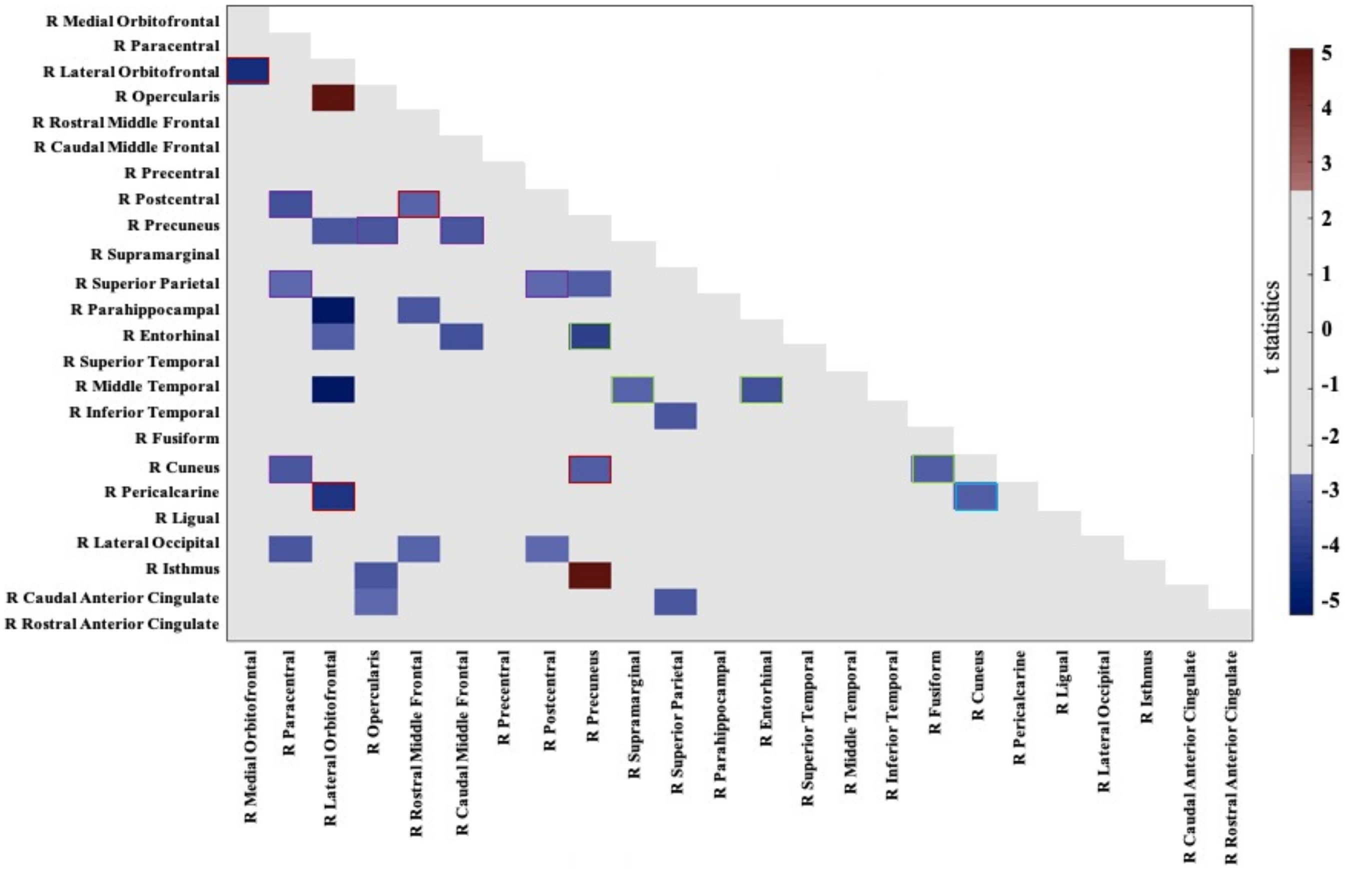


**Supplementary Figure 4. Graphical overview of coherence comparisons of ROIs in the left (R) hemisphere between the patient and controls.** Colors correspond to t statistics, blue indicates that the patient's connectivity values were significantly lower than those of the controls, red indicates that the patient's connectivity values were significantly higher than those of the controls. Non-significant differences are plotted in light gray. ROIs within inferior fronto occipital fasciculus (red), inferior longitudinal fasciculus (blue), superior longitudinal fasciculus (purple) were represented with rectangles. ROIs within both calcarine and inferior fronto-occipital fasciculus were represented with green rectangles.

**Supplementary Table 3. Resting state fMRI connectivity comparisons in the Inferior Fronto-Occipital Fasciculus (IFOF).** The table summarizes comparison of coherence scores between healthy participants and the patient for each ROI pairs along the IFOF. Given are the standard null hypothesis test (one -sample t-test) results with corresponding 99% confidence intervals that are derived from nonparametric bootstrapping procedure of healthy participants as well as the patient's raw coherence score in left (L) and right (R) hemispheres. Probability outcomes of each test were adjusted via false discovery rate (FDR; Benjamini and Hochberg, 1995) procedures and resulting $P_{FDR}$ values were reevaluated at a criterion of 0.05 (*), 0.01 (**) and 0.001 (***) for statistical significance. Results are shown in t test score, [confidence intervals], patient's raw score format.

| *IFOF* | *Lateral Orbitofrontal* | *Postcentral* | *Supramarginal* | *Precuneus* |
|---|---|---|---|---|
| *Rostral Middle Frontal* | **L:7.477***,[0.1031-0.2732],0.477**<br>R:3.278*,[0.1197-0.3426],0.394 | L:-1.673,[0.0806-0.4256],0.074<br>**R:-2.798*,[0.1009-0.4161],0.025** | L:-1.233,[0.1282-0.4816],0.181<br>R:-1.640,[0.1261-0.4002],0.131 | L:-1.385,[0.0990-0.4583],0.152<br>R:-1.898,[0.0953-0.4553],0.081 |
| *Medial Orbitofrontal* | L:-1.215,[0.0742-0.2649],0.108<br>**R:-4.077*,[0.0835-0.1742],0.037** | L:-1.924,[0.0915-0.3641],0.080<br>R:-0.791,[0.0694-0.3450],0.130 | L:-2.320,[0.0912-0.3187],0.068<br>R:-2.375,[0.0963-0.3206],0.044 | L:3.886**,[0.1003-0.4112],0.051<br>R:-1.240,[0.0959-0.4157],0.14 |
| *Caudal Middle Frontal* | L:-2.290,[0.1033-0.3279]0.084<br>R:2.174,[0.1442-0.3217],0.31 | L:-1.197,[0.0903-0.3775],0.124<br>R:-1.157, [0.128-0.4624], 0.102 | L:-1.253,[0.865-0.3809],0.118<br>R:-1.258,[0.1040-0.3776],0.131 | L:-0.614,[0.0975-0.4187],0.185<br>**R:-3.125*,[0.1209-0.41],0.035** |
| *Supramarginal* | L:0.905, [0.0589-0.2395],0.18<br>R:-1.389,[0.0697-0.1759], 0.83 | L:0.083,[0.1530-0.4808],0.302<br>R:-2.440,[0.1550-0.4760],0.112 | L:0.000<br>R:0.000 | L:-2.373,[0.1474-0.4446],0.109<br>R:-3.155*,[0.1450-0.4179],0.066 |
| *Pericalcarine* | L:3.241*,[0.0756-0.1991], 0.22<br>**R:-4.025**,[0.0971-0.1873],0.055** | L:-2.089,[0.1458-0.4338],0.117<br>R:-0.547,[0.1379-0.4101],0.2 | L:-1.853,[0.1039-0.39],0.093<br>R:-0.272,[0.093-0.3751],0.189 | **L:-3.269*, [0.1816-0.5041], 0.078**<br>R:-2.914*,[0.1626-0.4723],0.077 |
| *Cuneus* | L:1.827,[0.0647-0.1865],0.175<br>R:-2.525*,[0.0854-0.1656],0.074 | L:-2.492*, [0.1581-0.4551], 0.111<br>R:-2.967*,[0.1282-0.3828],0.049 | L:-2.069,[0.1114-0.3817],0.093<br>R:-1.733,[0.1161-0.3532],0.116 | **L:-3.101*, [0.1702-0.4998], 0.073**<br>**R:-2.886*,[0.1309-0.4314],0.047** |
| *Lateral Occipital* | L:-2.194,[0.0818-0.1904],0.074<br>R:-3.028*,[0.0752-0.1882],0.048 | **L:-3.078*,[0.1788-0.4302],0.089**<br>**R:-2.543*,[0.1637-0.4193],0.111** | L:-1.265,[0.0918-0.3259],0.111<br>R:-1.789,[0.1016-0.3545],0.077 | L:-2.485,[0.1530-0.4512],0.103<br>R:-2.002,[0.129-0.4456],0.111 |

**Supplementary Table 4. Resting state fMRI connectivity comparisons in the Superior Longitudinal Fasciculus (SLF)**. The table summarizes comparison of coherence scores between healthy participants and the patient for each ROI pairs along the SLF. Given are the standard null hypothesis test (one -sample t-test) results with corresponding 99% confidence intervals that are derived from nonparametric bootstrapping procedure of healthy participants as well as the patient's raw coherence score in left (L) and right (R) hemispheres. Probability outcomes of each test were adjusted via false discovery rate (FDR; Benjamini and Hochberg, 1995) procedures and resulting $P_{FDR}$ values were reevaluated at a criterion of 0.05 (*), 0.01 (**) and 0.001 (***) for statistical significance. Results are shown in t test score, [confidence intervals], patient's raw score format.

| *SLF* | *Caudal Middle Frontal* | *Precentral* | *Postcentral* | *Paracentral* | *Opercularis* |
|---|---|---|---|---|---|
| *Precentral* | L:-2.222,[0.0913-0.3570],0.068<br>R:-1.989,[0.108-0.3789],0.091 | L:0.000<br>R:0.000 | **L:-4.065**,[0.2672-0.5351],0.144**<br>R:-1.946,[0.2183-0.5428],0.23 | L:-2.256,[0.1581-0.4340],0.14<br>R:-1.719,[0.19-0.4752],0.208 | L:-1.574,[0.1236-0.4166],0.143<br>R:-1.189,[0.1502-0.4099],0.177 |
| *Paracentral* | L:-0.748,[0.0707-0.3875],0.133<br>R:-2.171,[0.0755-0.3624],0.046 | L:-2.256,[0.1545-0.433],0.14<br>R:-1.719,[0.19-0.4752],0.208 | L:-1.857,[0.1687-0.4064],0.19<br>**R:-3.244*,[0.1531-0.4665],0.056** | L:0.000<br>R:0.000 | L:-1.792,[0.086-0.3936],0.07<br>R:-2.138,[0.0851-0.436],0.043 |
| *Superior Parietal* | L:-1.930,[0.1135-0.4733],0.113<br>R:-0.629, [0.1157-0.4877], 0.113 | **L:-3.221*,[0.1341-0.4545],0.071**<br>R:-3.019*,[0.1439-0.4295],0.072 | **L:-3.431**, [0.1157-0.4903],0.034**<br>**R:-2.643*,[0.1135-0.4517],0.051** | L:-1.666,[0.061-0.3748],0.039<br>**R:-2.552*,[0.1002-0.3885],0.040** | L:-0.612,[0.1916-0.4983],0.254<br>R:0.159,[0.2308-0.5129],0.36 |
| *Supramarginal G.* | L:-1.253,[0.865-0.3809],0.118<br>R:-1.258,[0.1040-0.3776],0.131 | **L:-2.690*,[0.1229-0.457],0.05**<br>R:-1.225,[0.1418-0.4314],0.177 | L:0.083,[0.1530-0.4808],0.302<br>R:-2.440,[0.1550-0.4760],0.112 | L:-0.889,[0.0725-0.3982],0.129<br>R:-1.264,[0.0977-0.3839],0.125 | L:-2.207,[0.115-0.4669],0.083<br>R:-2.461*,[0.1477-0.4628],0.097 |
| *Precuneus* | L:-0.614,[0.0975-0.4187],0.185<br>**R:-3.125*,[0.1209-0.41],0.035** | **L:-3.948**,[0.1907-0.4483],0.066**<br>R:-2.450*,[0.1538-0.4157],0.119 | **L:-3.371*, [0.1690-0.4779], 0.051**<br>R:-2.750*,[0.1275-0.4556],0.063 | L:-0.201,[0.0845-0.3953],0.185<br>R:-0.455,[0.0929-0.4197],0.192 | L:-1.733,[0.0844-0.4123], 0.073<br>**R:-3.091*,[0.128-0.4624], 0.03** |
| *Pericalcarine* | L:-2.476*,[0.0778-0.4208],0.0144<br>R:-0.916,[0.1551-0.428],0.187 | **L:-3.897**,[0.1838-0.4567],0.062**<br>R:-0.164,[0.1687-0.4156],0.26 | L:-2.089,[0.1458-0.4338],0.117<br>R:-0.547,[0.1379-0.4101],0.2 | L:-1.067,[0.119-0.4324],0.145<br>R:-2.294,[0.1372-0.466],0.08 | L:-1.568,[0.0829-0.4072],0.074<br>R:-0.550,[0.0848-0.4203],0.154 |
| *Lingual G.* | L:-2.791*,[0.1156-0.4028],0.048<br>R:-1.009,[0.1326-0.4252],0.177 | L:-3.941**,[0.1826-4388],0.075<br>R:-0.721,[0.1594-0.4392],0.241 | L:-2.354,[0.1455-0.4013],0.123<br>R:-1.536,[0.1375-0.4085],0.156 | L:-1.255,[0.1053-0.3507],0.124<br>R:-1.851,[0.1417-0.4106],0.136 | L:-1.806,[0.0877-0.3704],0.076<br>R:-1.245,[0.1191-0.3985],0.133 |
| *Cuneus* | L:-2.376,[0.0825-0.3838],0.029<br>R:-1.475,[0.1354-0.3763],0.141 | **L:-4.995**, [0.2150-0.4444], 0.063**<br>R:-2.932*,[0.1781-0.3928],0.131 | L:-2.492*, [0.1581-0.4551], 0.111<br>R:-2.967*,[0.1282-0.3828],0.049 | L:-1.699,[0.1328-0.4311],0.119<br>**R:-3.074*,[0.1447-0.4212],0.048** | L:-1.748,[0.0914-0.3917],0.082<br>R:-0.669,[0.0779-0.3686],0.138 |

**Supplementary Table 5. Resting state fMRI connectivity in the Inferior Longitudinal Fasciculus (ILF).** The table summarizes comparison of coherence scores between healthy participants and the patient for each ROI pairs along the ILF. Given are the standard null hypothesis test (one -sample t-test) results with corresponding 99% confidence intervals that are derived from nonparametric bootstrapping procedure of healthy participants as well as the patient's raw coherence score in left (L) and right (R) hemispheres. Probability outcomes of each test were adjusted via false discovery rate (FDR; Benjamini and Hochberg, 1995) procedures and resulting $P_{FDR}$ values were reevaluated at a criterion of 0.05 (*), 0.01 (**) and 0.001 (***) for statistical significance. Results are shown in t test score, [confidence intervals], patient's raw score format.

| *ILF* | *Entorhinal* | *Fusiform* | *Parahippocampal G.* | *Supramarginal G.* | *Pericalcarine* |
|---|---|---|---|---|---|
| *Superior Temporal* | L:1.020,[0.0889-0.3475],0.239<br>R:-2.021,[0.0723-0.2402],0.053 | L:2.151,[0.0624-0.2574],0.238<br>R:-1.423,[0.0766-0.3826],0.082 | L:-1.149,[0.0876-0.2679],0.106<br>R:-2.449,[0.1109-0.2352], 0.097 | L:-0.694,[0.0728-0.3842],0.148<br>R:-1.383, [0.061-0.3685], 0.111 | L:-1.761,[0.119-0.4505],0.09<br>R:-2.15,[0.0988-0.4301],0.049 |
| *Middle Temporal* | L:0.027,[0.0596-0.2769],0.135<br>**R:-3.246*,[0.0766-0.2072],0.035** | L:4.274**,[0.0813-0.2774],0.357<br>R:-1.314,[0.0634-0.3658],0.075 | L:-1.324,[0.0642-0.2911],0.08<br>R:-0.471,[0.083-0.2206],0.117 | L:-2.864*,[0.1152-0.3404],0.056<br>**R:-2.741*,[0.098-0.3458],0.025** | L:-1.575,[0.0676-0.3558],0.054<br>R:-2.009,[0.0867-0.406],0.035 |
| *Inferior Temporal* | L:-1.393,[0.0592-0.2268],0.056<br>R:-2.026,[0.0616-0.2236],0.062 | L:-1.101,[0.0834-0.3406],0.118<br>R-:0.56,[0.1234-0.4075],0.201 | L:-1.053,[0.0544-0.2406],0.074<br>R:0.434,[0.0632-0.2431],0.149 | **L:-3.420**,[0.1114-0.2704],0.053**<br>R:-0.910,[0.0936-0.3994],0.154 | L:-2.467*,[0.1035-0.2976],0.076<br>R:-0.346,[0.1426-0.4864],0.261 |
| *Precuneus* | L:1.173,[0.0815-0.3211],0.243<br>**R:-3.681**,[0.1049-0.2829]0.033** | L:5.348***,[0.0867-0.2824], 0.415<br>R:-0.964,[0.1139-0.3685],0.15 | L:-2.129,[0.1067-0.2961],0.098<br>R:-1.894,[0.1151-0.3407],0.134 | L:-2.373,[0.1474-0.4446],0.109<br>R:-3.155*,[0.1450-0.4179],0.066 | **L:-3.269*, [0.1816-0.5041], 0.078**<br>R:-2.914*,[0.1626-0.4723],0.077 |
| *Cuneus* | **L:-2.725*, [0.1064-0.3293], 0.044**<br>R:-2.527,[0.1135-0.2740],0.084 | L:-1.296,[0.0728-0.2745],0.085<br>**R:-2.910*,[0.1436-0.4048],0.078** | L:2.118,[0.0885-0.2394],0.218<br>R:-3.619**,[0.1364-0.2453],0.095 | L:-2.069,[0.1114-0.3817],0.093<br>R:-1.733,[0.1161-0.3532],0.116 | L:1.637,[0.397-0.6467],0.602<br>**R:-2.824*,[0.4198-0.6588],0.360** |
| *Lingual G.* | L:-1.459,[0.0892-0.2948],0.094<br>R:-2.458*,[0.1069-0.2626],0.093 | L:-0.662,[0.075-0.3101],0.139<br>R:-1.637,[0.1189-0.4258],0.128 | L:1.899,[0.0719-0.2576],0.231<br>R:0.447,[0.1332-0.3306],0.249 | L:-2.447,[0.0972-0.3481],0.058<br>R:-1.182,[0.1522-0.3837],0.182 | L:2.139,[0.4562-0.6603],0.659<br>R:-1.391,[0.3454-0.6767],0.411 |
| *Lateral Occipital* | L:2.473*,[0.0941-0.3010],0.052<br>R:-1.302,[0.0989-0.2667],0.121 | L:-1.159,[0.0704-0.2776],0.092<br>R:-2.081,[0.089-0.3503],0.05 | L:-0.863,[0.069-0.2426],0.104<br>R:-0.198,[0.1306-0.248],0.178 | L:-1.265,[0.0918-0.3259],0.111<br>R:-1.789,[0.1016-0.3545],0.077 | L:-2.228,[0.2955-0.5912],0.279<br>R:-0.071,[0.3209-0.6409],0.471 |

**Supplementary Table 6. Resting state fMRI connectivity comparisons in the calcarine.** The table summarizes comparison of coherence scores between healthy participants and the patient for each ROI pairs along calcarine tracts. Given are the standard null hypothesis test (one -sample t-test) results with corresponding 99% confidence intervals that are derived from nonparametric bootstrapping procedure of healthy participants as well as the patient's raw coherence score in left (L) and right (R) hemispheres. Probability outcomes of each test were adjusted via false discovery rate (FDR; Benjamini and Hochberg, 1995) procedures and resulting $P_{FDR}$ values were reevaluated at a criterion of 0.05 (*), 0.01 (**) and 0.001 (***) for statistical significance. Results are shown in t test score, [confidence intervals], patient's raw score format.

| *CALCARINE* | *Precuneus* | *Pericalcarine* |
|---|---|---|
| *Pericalcarine* | **L:-3.269*, [0.1816-0.5041], 0.078**<br>R:-2.914*,[0.1626-0.4723],0.077 | L:0.000<br>R:0.000 |
| *Cuneus* | **L:-3.101*, [0.1702-0.4998], 0.073**<br>**R:-2.886*,[0.1309-0.4314],0.047** | L:1.637,[0.397-0.6467],0.602<br>**R:-2.824*,[0.4198-0.6588],0.360** |
| *Lingual G* | L:-3.035*,[0.18-0.4493],0.118<br>R:-1.977,[0.1549-0.4556],0.156 | L:2.139,[0.4562-0.6603],0.659<br>R:-1.391,[0.3454-0.6767],0.411 |

**Supplementary Table 7. Resting state fMRI connectivity comparisons in the cingulate.** The table summarizes comparison of coherence scores between healthy participants and the patient for each ROI pairs along the cingulate tracts. Given are the standard null hypothesis test (one -sample t-test) results with corresponding 99% confidence intervals that are derived from nonparametric bootstrapping procedure of healthy participants as well as the patient's raw coherence score in left (L) and right (R) hemispheres. Probability outcomes of each test were adjusted via false discovery rate (FDR; Benjamini and Hochberg, 1995) procedures and resulting $P_{FDR}$ values were reevaluated at a criterion of 0.05 (*), 0.01 (**) and 0.001 (***) for statistical significance. Results are shown in t test score, [confidence intervals], patient's raw score format.

| *CINGULATE* | *Caudate Anterior Cingulate* |
|---|---|
| *Isthmus* | L:-2.385,[0.0882-0.3418],0.042<br>R:-2.467*,[0.0881-0.3085],0.055 |
| *Entorinal* | L:-1.550,[0.1115-0.3359],0.112<br>R:--2.582*,[0.0752-0.2463],0.048 |
| *Rostral Anterior Cingulate* | L:-1.781,[0.1002-0.3789],0.107<br>R:2.328,[0.0886-0.4291],0.037 |

**Supplementary Table 8. Resting state fMRI connectivity comparisons in the tapetum.** The table summarizes comparison of coherence scores between healthy participants and the patient for each ROI pairs along the cingulate tracts. Given are the standard null hypothesis test (one -sample t-test) results with corresponding 99% confidence intervals that are derived from nonparametric bootstrapping procedure of healthy participants as well as the patient's raw coherence score in left (L) and right (R) hemispheres. Probability outcomes of each test were adjusted via false discovery rate (FDR; Benjamini and Hochberg, 1995) procedures and resulting $P_{FDR}$ values were reevaluated at a criterion of 0.05 (*), 0.01 (**) and 0.001 (***) for statistical significance. Results are shown in t test score, [confidence intervals], patient's raw score format.

| *TAPETUM* | *R Cuneus* | *R Lateral Occipital* |
|---|---|---|
| *L Cuneus* | -2.733*,[0.4569-0.7048], 0.416 | 1.288,[0.2691-0.5651],0.474 |
| *L Lateral Occipital* | -1.615,[0.2794-0.5714],0.301 | -3.831,[0.4511-0.6996],0.347 |

**Supplementary Table 9a. Raw coherence scores of the participants that are used for functional connectivity comparison in Supplementary Table 3.** Given are minimum and maximum boundaries of raw coherence scores in controls and raw coherence scores of the patient for each ROI pairs along adjacent gray matter regions of major white matter tracts, namely IFOF in left (L) and right (R) hemispheres. Significantly differences are written in bold.

| *IFOF* | *Lateral Orbitofrontal* | *Postcentral* | *Supramarginal* | *Precuneus* |
|---|---|---|---|---|
| *Rostral Middle Frontal* | **L:0.051-0.383],0.477**<br>R:0.0511-0.5369,0.394 | L:0.053-0.8124,0.074<br>**R:0.0609-0.7295,0.025** | L:0.0744-0.8013,0.181<br>R:0.0812-0.6941,0.131 | L:0.03-0.7303,0.152<br>R:0.0435-0.8484,0.081 |
| *Medial Orbitofrontal* | L:0.03-0.391,0.108<br>**R:0.0391-0.2666,0.037** | L:0.0369-0.65,0.080<br>R:0.0237-0.6409,0.130 | L:0.0286-0.5358,0.068<br>R:0.0688-0.6183,0.044 | L:0.0314-0.7566,0.051<br>R:0.034-0.7177,0.14 |
| *Caudal Middle Frontal* | L:0.0555-0.5026,0.084<br>R:0.1178-0.4757,0.31 | L:0.0554-0.6906,0.124<br>R:0.0656-0.6841, 0.102 | L:0.0464-0.6919,0.118<br>R:0.0332-0.675,0.131 | L:0.0324-0.7533,0.185<br>**R:0.0595-0.7029,0.035** |
| *Supramarginal* | L:0.0155-0.4094,0.18<br>R:0.0186-0.2726,0.083 | L:0.0636-0.7173,0.302<br>R:0.086-0.77260,0.112 | L:0.000<br>R:0.000 | L:0.0957-0.7214,0.109<br>R:0.408-0.6652,0.066 |
| *Pericalcarine* | L:0.0602-0.319, 0.22<br>**R:0.0561-0.2369,0.055** | L:0.1025-0.7483,0.117<br>R:0.0853-0.7189,0.2 | L:0.0734-0.6874,0.093<br>R:0.0587-0.6707,0.189 | **L:0.101-0.799, 0.078**<br>R:0.062-0.8122,0.077 |
| *Cuneus* | L:0.0289-0.2761,0.175<br>R:0.047-0.2369,0.074 | L:0.0642-0.7646, 0.111<br>R:0.034-0.6803,0.049 | L:0.0694-0.6505,0.093<br>R:0.0488-0.6062,0.116 | **L:0.1151-0.817, 0.073**<br>**R:0.0491-0.7743,0.047** |
| *Lateral Occipital* | L:0.0596-0.2788,0.074<br>R:0.0279-0.29,0.048 | **L:0.1318-0.7344,0.089**<br>**R:0.1129-0.7121,0.111** | L:0.0615-0.5969,0.111<br>R:0.0711-0.6545,0.077 | L:0.084-0.768,0.103<br>R:0.0442-0.7783,0.111 |

**Supplementary Table 9b. Raw coherence scores of the participants that are used for functional connectivity comparison in Supplementary Table 4.** Given are minimum and maximum boundaries of raw coherence scores in controls and raw coherence scores of the patient for each ROI pairs along adjacent gray matter regions of major white matter tracts, namely SLF in left (L) and right (R) hemispheres. Significantly differences are written in bold.

| *SLF* | *Caudal Middle Frontal* | *Precentral* | *Postcentral* | *Paracentral* | *Opercularis* |
|---|---|---|---|---|---|
| *Precentral* | L:0.0422-0.6007,0.068<br>R:0.0788-0.6535,0.091 | L:0.000<br>R:0.000 | **L:0.1823-0.7053,0.144**<br>R:0.1084-0.6983,0.23 | L:0.0548-0.6669,0.14<br>R:0.1121-0.6577,0.208 | L:0.0522-0.6763,0.143<br>R:0.1116-0.7021,0.177 |
| *Paracentral* | L:0.039-0.7484,0.133<br>R:0.0563-0.6347,0.046 | L:0.0548-0.6669,0.14<br>R:0.1121-0.6577,0.208 | L:0.0676-0.869,0.19<br>**R:0.0874-0.6985,0.056** | L:0.000<br>R:0.000 | L:0.0602-0.7422,0.07<br>R:0.0373-0.8206,0.043 |
| *Superior Parietal* | L:0.0501-0.79120.,0.113<br>R:0.0501-0.7912, 0.113 | **L:0.1060-0.7119,0.071**<br>R:0.071-0.6636,0.072 | **L:0.0694-0.8175,0.034**<br>**R:0.0643-0.7759,0.051** | L:0.045-0.7571,0.039<br>**R:0.0673-0.6991,0.040** | L:0.139-0.8545,0.254<br>R:0.124-0.8254,0.36 |
| *Supramarginal G.* | L:0.0464-0.6919,0.118<br>R:0.0332-0.675,0.131 | **L:0.0814-0.6841,0.05**<br>R:0.0597-0.7324,0.177 | L:0.0636-0.7173,0.302<br>R:0.086-0.7726,0.112 | L:0.0448-0.7108,0.129<br>R:0.0412-0.7023,0.125 | L:0.04-0.797,0.083<br>R:0.0933-0.7724,0.097 |
| *Precuneus* | L:0.0324-0.7533,0.185<br>**R:0.0595-0.7029,0.035** | **L:0.1299-0.681,0.066**<br>R:0.0506-0.6201,0.119 | **L:0.1116-0.7944, 0.051**<br>R:0.0227-0.7183,0.063 | L:0.0394-0.7963,0.185<br>R:0.0304-0.7565,0.192 | L:0.0315-0.7911, 0.073<br>**R:0.0344-0.7863, 0.03** |
| *Pericalcalcarine* | L:0.0308-0.7785,0.0144<br>R:0.0973-0.7425,0.187 | **L:0.0817-0.6982,0.062**<br>R:0.0859-0.6627,0.26 | L:0.1025-0.7483,0.117<br>R:0.0853-0.7189],0.2 | L:0.0517-0.8057,0.145<br>R:0.0709-0.8449,0.08 | L:0.0112-0.7995,0.074<br>R:0.0443-0.8273,0.154 |
| *Lingual G.* | L:0.0391-0.706,0.048<br>R:0.0727-0.7634,0.177 | L:0.0581-0.577,0.075<br>R:0.048-0.6626,0.241 | L:0.0442-0.6247,0.123<br>R:0.0424-0.7044,0.156 | L:0.0482-0.6347],0.124<br>R:0.0697-0.7017,0.136 | L:0.0211-0.6928,0.076<br>R:0.0832-0.7263,0.133 |
| *Cuneus* | L:0.0342-0.7329,0.029<br>R:0.0653-0.6698,0.141 | **L:0.109-0.6339, 0.063**<br>R:0.091-0.5866,0.131 | L:0.0642-0.7646, 0.111<br>R:0.034-0.6803,0.049 | L:0.0635-0.7834,0.119<br>**R:0.0777-0.7594,0.048** | L:0.0356-0.755,0.082<br>R:0.0452-0.7189,0.138 |

**Supplementary Table 9c. Raw coherence scores of the participants that are used for functional connectivity comparison in Supplementary Table 5.** Given are minimum and maximum boundaries of raw coherence scores in controls and raw coherence scores of the patient for each ROI pairs along adjacent gray matter regions of major white matter tracts, namely ILF in left (L) and right (R) hemispheres. Significantly differences are written in bold.

| *ILF* | *Entorhinal* | *Fusiform* | *Parahippocampal G.* | *Supramarginal G.* | *Pericalcarine* |
|---|---|---|---|---|---|
| *Superior Temporal* | L:0.0627-0.6589,0.239<br>R:0.0417-0.4366,0.053 | L:0.0368-0.484,0.238<br>R:0.0559-0.7487,0.082 | L:0.0596-0.4805,0.106<br>R:0.06-0.3487, 0.097 | L:0.055-0.7004,0.148<br>R:0.0271-0.6798, 0.111 | L:0.0636-0.8576,0.09<br>R:0.0607-0.8133,0.049 |
| *Middle Temporal* | L:0.0366-0.542,0.135<br>**R:0.0473-0.3233,0.035** | L:0.0532-0.5170,0.357<br>R:0.035-0.7296,0.075 | L:0.0304-0.5557,0.08<br>R:0.0625-0.3984,0.117 | L:0.0469-0.5783,0.056<br>**R:0.0676-0.6451,0.025** | L:0.0391-0.7111,0.054<br>R:0.0612-0.7988,0.035 |
| *Inferior Temporal* | L:0.0319-0.4345,0.056<br>R:0.036-0.2981,0.062 | L:0.0442-0.5944,0.118<br>R:0.0708-0.706,0.201 | L:0.15-0.4584,0.074<br>R:0.0282-0.4644,0.149 | **L:0.0595-0.4241,0.053**<br>R:0.0633-0.7154,0.154 | L:0.0385-0.4933,0.076<br>R:0.0664-0.8217,0.261 |
| *Precuneus* | L:0.0505-0.5869,0.243<br>**R:0.0726-0.4267,0.033** | L:0.0595-0.4631, 0.415<br>R:0.0727-0.6691,0.15 | L:0.0337-0.4767,0.098<br>R:0.0299-0.4068,0.134 | L:0.0957-0.7214,0.109<br>R:0.0408-0.6652,0.066 | **L:0.101-0.799, 0.078**<br>R:0.062-0.8122,0.077 |
| *Cuneus* | **L:0.0672-0.5983, 0.044**<br>R:0.0647-0.4535,0.084 | L:0.0577-0.5052,0.085<br>**R:0.0818-0.6688,0.078** | L:0.0515-0.4118,0.218<br>R:0.0927-0.3229,0.095 | L:0.0694-0.6505,0.093<br>R:0.0488-0.6062,0.116 | L:0.2947-0.8945,0.602<br>**R:0.3811-0.8815,0.36** |
| *Lingual G.* | L:0.0554-0.5401,0.094<br>R:0.0332-0.3797,0.093 | L:0.0385-0.5172],0.139<br>R:0.0608-0.7435,0.128 | L:0.0327-0.4685,0.231<br>R:0.0591-0.4192,0.249 | L:0.0588-0.5821,0.058<br>R:0.0991-0.6246,0.182 | L:0.2871-0.8075,0.659<br>R:0.1793-0.8772,0.411 |
| *Lateral Occipital* | **L:0.0522-0.536,0.052**<br>R:0.0716-0.4237,0.121 | L:0.0526-0.5083,0.092<br>R:0.059-0.6834,0.05 | L:0.0334-0.4218,0.104<br>R:0.0896-0.3265,0.178 | L:0.0615-0.5969,0.111<br>R:0.0711-0.6545,0.077 | L:0.1604-0.875,0.279<br>R:0.1801-0.8434,0.471 |

**Supplementary Table 9d. Raw coherence scores of the participants that are used for functional connectivity comparison in Supplementary Table 6.** Given are minimum and maximum boundaries of raw coherence scores in controls and raw coherence scores of the patient for each ROI pairs along adjacent gray matter regions of major white matter tracts, namely calcarine in left (L) and right (R) hemispheres. Significantly differences are written in bold.

| *CALCARINE* | *Precuneus* | *Pericalcarine* |
|---|---|---|
| *Pericalcarine* | **L:0.101-0.799, 0.078**<br>R:0.062-0.8122,0.077 | L:0.000<br>R:0.000 |
| *Cuneus* | **L:0.1151-0.817], 0.073**<br>**R:0.0491-0.7743,0.047** | L:0.0247-0.8945,0.602<br>**R:0.3811-0.8815,0.360** |
| *Lingual G* | L:0.0742-0.678,0.118<br>R:0.0305-0.7238,0.156 | L:0.2871-0.8075,0.659<br>R:0.1793-0.8772,0.411 |

**Supplementary Table 9e. Raw coherence scores of the participants that are used for functional connectivity comparison in Supplementary Table 7.** Given are minimum and maximum boundaries of raw coherence scores in controls and raw coherence scores of the patient for each ROI pairs along adjacent gray matter regions of major white matter tracts, namely cingulate in left (L) and right (R) hemispheres. Significantly differences are written in bold.

| *CINGULATE* | *Caudate Anterior Cingulate* |
|---|---|
| *Isthmus* | L:0.0262-0.6244,0.042<br>R:0.0595-0.4763,0.055 |
| *Entorinal* | L:0.0548-0.5926,0.112<br>R:0.0251-0.4046,0.048 |
| *Rostral anterior* | L:0.0407-0.6391,0.107<br>R:0.0381-0.7835,0.037 |

**Supplementary Table 9f. Raw coherence scores of the participants that are used for functional connectivity comparison in Table 1.** Given are minimum and maximum boundaries of raw coherence scores in controls and raw coherence scores of the patient for each ROI pairs along adjacent gray matter regions of major white matter tracts, namely tapetum in left (L) and right (R) hemispheres. Significantly differences are written in bold.

| *TAPETUM* | *R Cuneus* | *R Lateral Occipital* |
|---|---|---|
| *L Cuneus* | 0.1638-0.9048, 0.417 | 0.192-0.8972,0.474 |
| *L Lateral Occipital* | 0.1865-0.9008, 0.316 | 0.228-0.91,0.347 |

***Functional Connectivity-Structural Connectivity Comparisons***

Radial diffusivity along left ILF and left supramarginal gyrus-inferior temporal functional connectivity r(33)=-0.7739,p=0.0086, radial diffusivity along right ILF and right superior temporal-entorhinal functional connectivity r(33)=-0.6891,p=0.0275, right superior temporal-pericalcarine r(33)=-0.669,p=0.0352 functional connectivity were positively correlated. Radial diffusivity along right ILF and right precuneus-supramarginal r(33)=-0.633,p=0.0495, right pericalcarine-precuneus r(33)=-0.7352,p=0.0154, right cuneus entorhinal r(33)=-0.6367,p=0.0477, right pericalcarine-cuneus r(33)=-0.6329,p=0.0495, right lingual-entorhinal r(33)=-0.6524,p=0.0409, right lateral occipital-entorhinal r(33)=-0.641,p=0.0458, right lateral occipital-supramarginal r(35)=-0.6702,p=0.034 functional connectivity were negatively correlated.

Mean diffusivity along left IFOF and left rostral middle frontal-lateral orbitofrontal functional connectivity r(26)=0.8448, p=0.0021was positively correlated. Radial diffusivity along left IFOF and rostral middle frontal-lateral orbitofrontal r(26)=0.7458, p=0.0133, left precuneus- medial orbitofrontal r(26)=0.6334, p=0.0493 were positively correlated. Fractional anisotropy along the right IFOF and right caudal middle frontal-lateral orbitofrontal functional connectivity r(26)=0.6329, p=0.0495 were marginally correlated. Radial diffusivity along the right IFOF and pericalcarine-lateral orbitofrontal r(26)=-0.6467, p=0.0433 was negatively correlated. In addition, axial diffusion along tapetum and the left and right cuneus functional connectivity r(26)=-0.6457, p=0.0437 was negatively correlated.

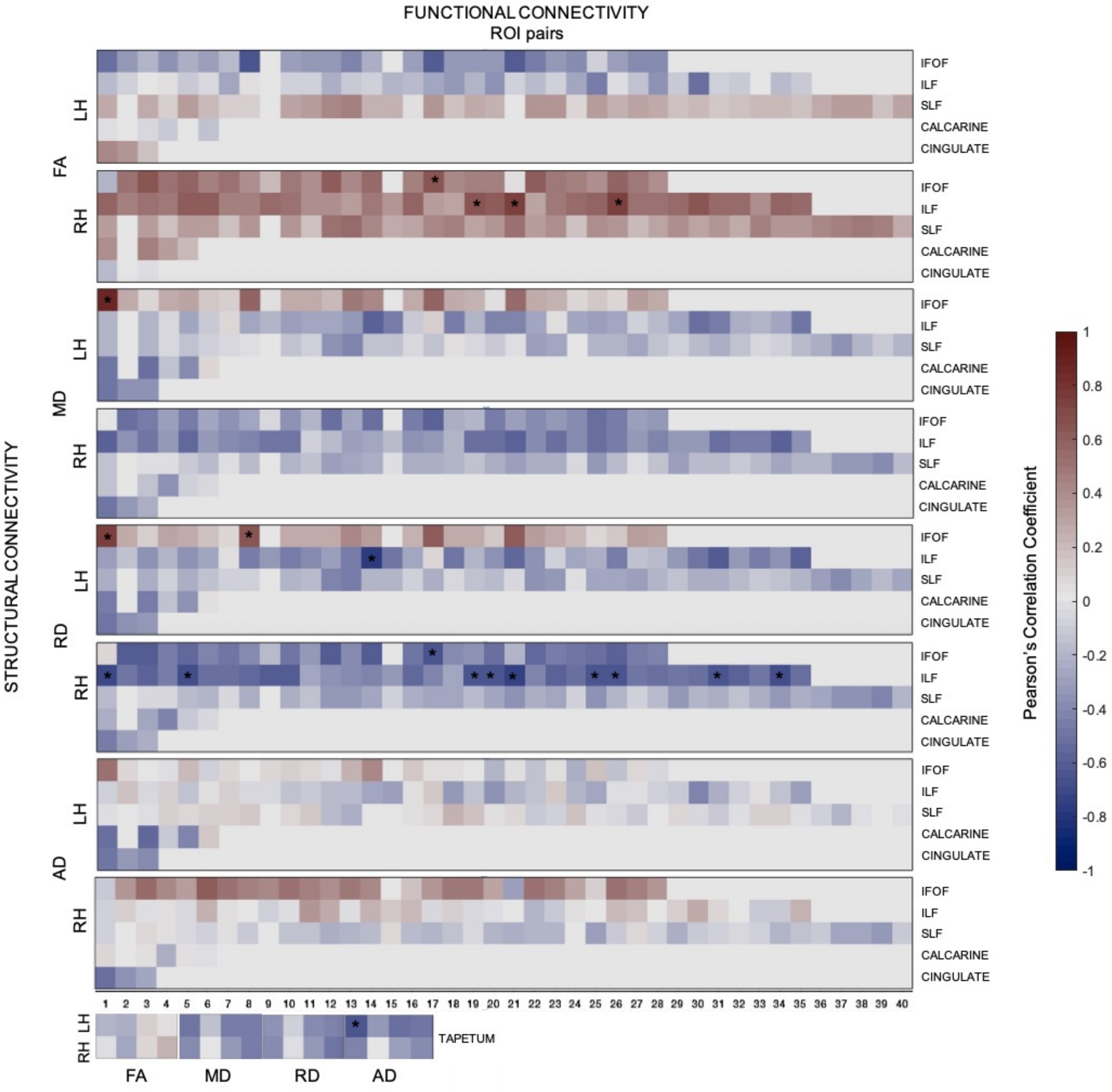


**Supplementary Figure 5. Comparison of functional and structural connectivity between the patient and controls.** Pearson's correlation coefficient was calculated between diffusion tensor scalars of white matter tracts of interests and functional connectivity of both intra- and interhemispheric region-of-interest pairs. Warm colors represent positive correlation coefficients whereas cold colors represent negative correlation coefficients. Statistical comparisons that correspond $p<0.05$ were indicated with asterisk. We found a significant positive correlation between functional and structural connectivity in the right ILF.
