# Supplementary material for: Cortical connectivity in the face of congenital structural changes—A case of homozygous LAMC3 mutation
Source: Brain Behav. 2021 Jun 14;11(8):e2241. doi: 10.1002/brb3.2241 (PMC8413815; doi:10.1002/brb3.2241)
Supplement: Supplementary file 2 — SUPPORTING INFORMATION [file BRB3-11-e2241-s002.docx]

**SUPPLEMENTARY ATLAS**

WM structural atlas comparison showed reduced fibers in bilateral IFOF, ILF, SLF and posterior corona radiata as well as the increased fibers in anterior corona radiata in the patient.

***Association Fibers*** Association fibers connect various parts of cerebral cortex and lie along anterior-posterior axis bilaterally. Two of the main WM connections in anterior-posterior axis- inferior fronto-occipital fasciculus (IFOF) and inferior longitudinal fasciculus (ILF) connects ipsilateral frontal areas to occipital, posterior parietal and temporal areas; and connects ipsilateral temporal areas to occipital areas, respectively (Wakana et al., 2004). In the inferior slices, Figure 1 A-B and C-D, reduced fibers in IFOF and ILF were identified in the patient compared to the control. Furthermore, reduction of fibers in other main WM connection superior longitudinal fasciculus (SLF) that connects fronto-temporal and fronto-parietal areas was revealed in patient (Figure 1 G-H, Figure 2 C-D and E-F).

***Projection Fibers*** Projection fibers that lie along the superior-inferior axis connect cerebral cortex with thalamus, pons and spinal cord (Wakana et al., 2004). Abundant and spread corona radiata fibers in the patient were identified in the superior parts (Figure 2 A-B, and C-D). Moreover, reduced white matter fibers in posterior corona radiata were identified in the patient (Figure 2 G-H).

White matter atlas comparison showed abnormal white matter structure in association fibers including IFOF, ILF, SLF and superior part of corona radiata in patient’s brain. To be able to reveal these possible alterations we next investigated white matter integrity and functional connectivity between cortical areas.

Label Glossary: ac-anterior comissure, acr-anterior region of corona radiata, alic-anterior limb of internal capsule, cbt-corticobulbar tract, cg-cingulum, cst-corticospinal tract, dscp-decussation of superior cerebral peduncle, icp-inferior cerebral peduncle,, ifo-inferior fronto-occipital fasciculus, ilf-inferior longitudinal fasciculus, pcr-posterior region of corona radiata, plic-posterior limb of internal capsule, scr-superior region of internal capsule, sfo-superior fronto-occipital fasciculus, slf-superior longitudinal fasciculus, unc-uncinate fasciculus.

Colors: red, green and blue represent fibers running along right-left, anterior-posterior, and superior-inferior axes, respectively, all images follow radiological conversion.


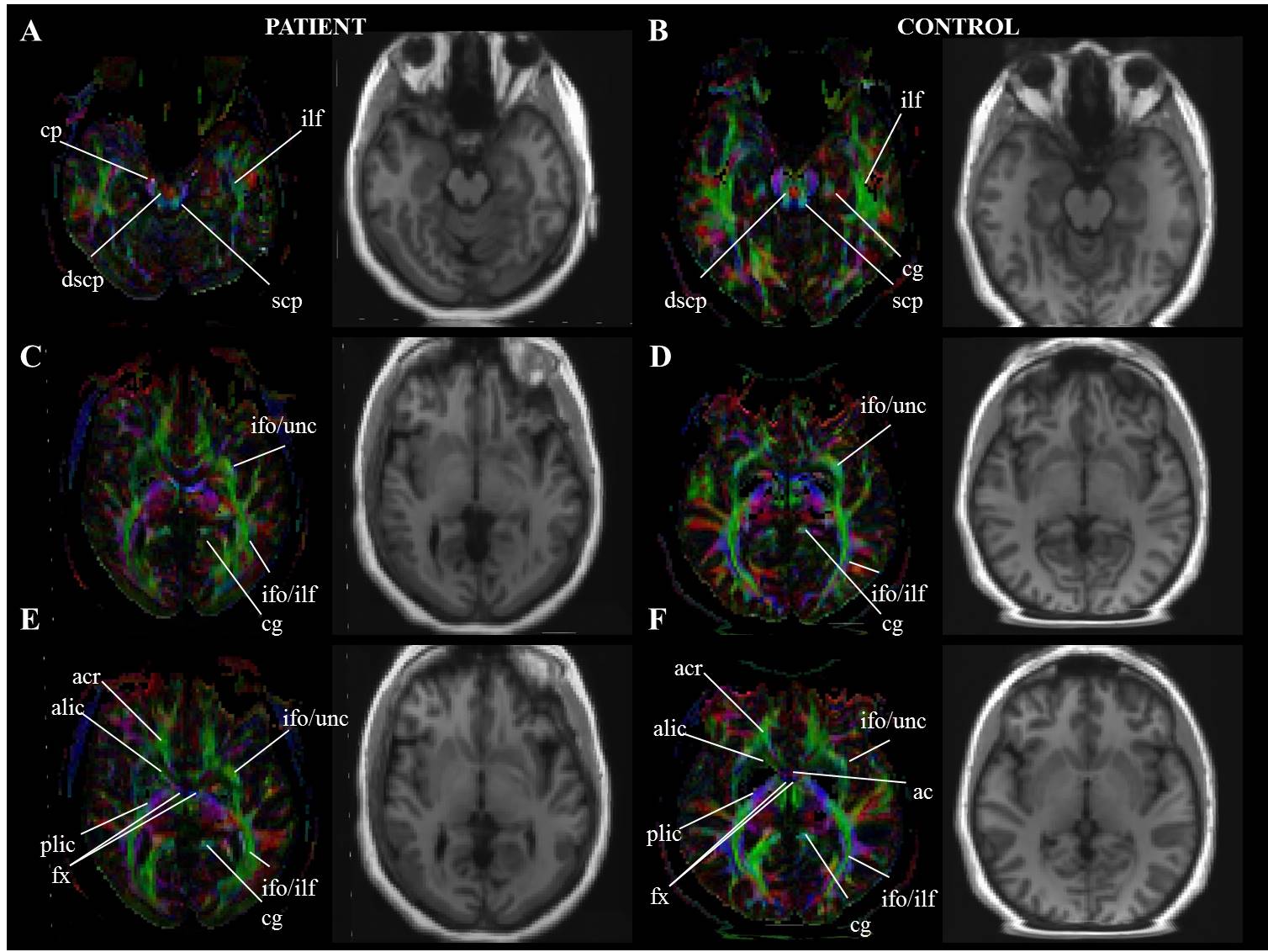

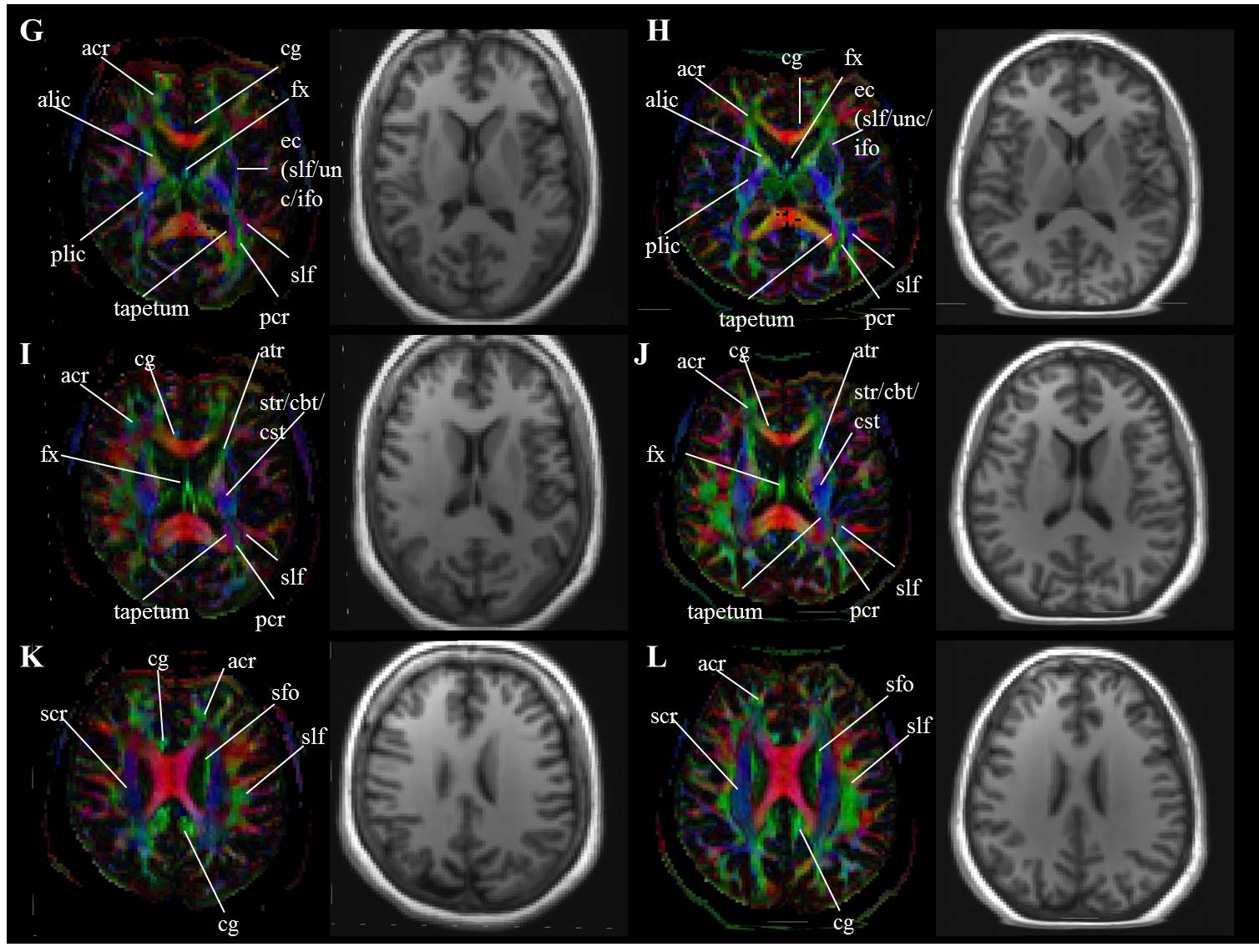

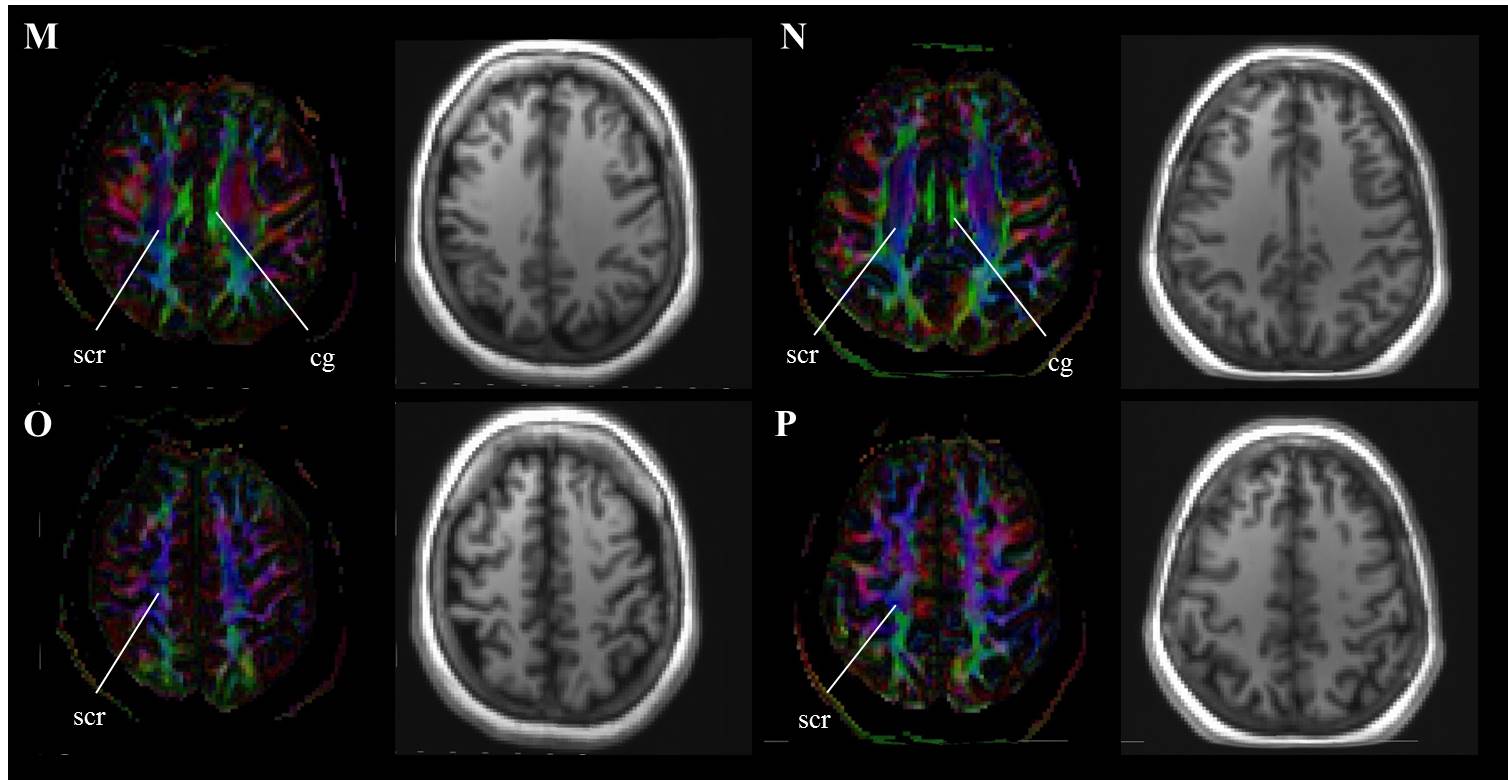


**Figure 1.** 2D axial DT imaging color maps and their corresponding T1 background images showing projections of brain stem, projection and association fibers on patient’s (first and second columns) and a representative control (third and fourth columns) data.


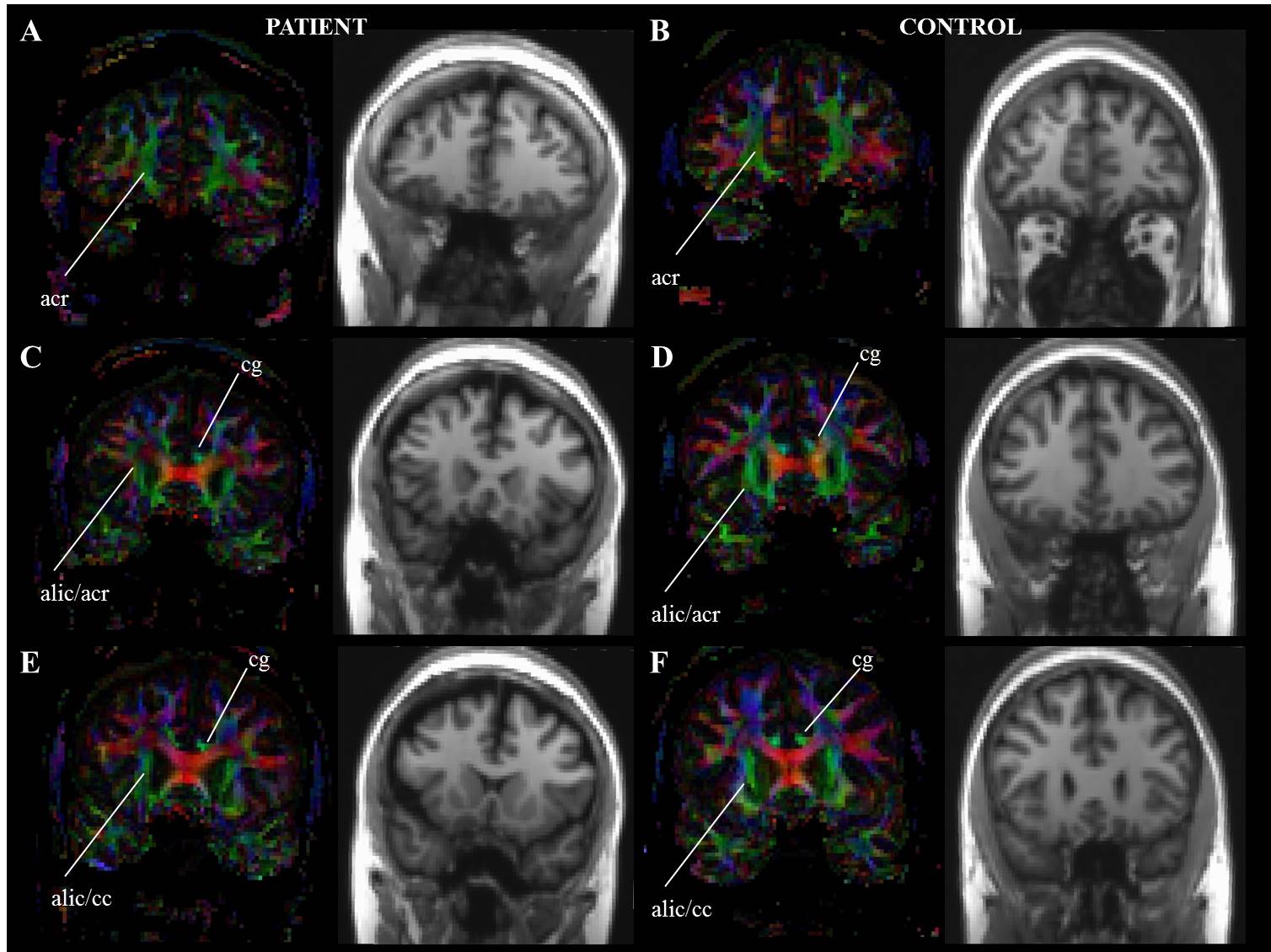

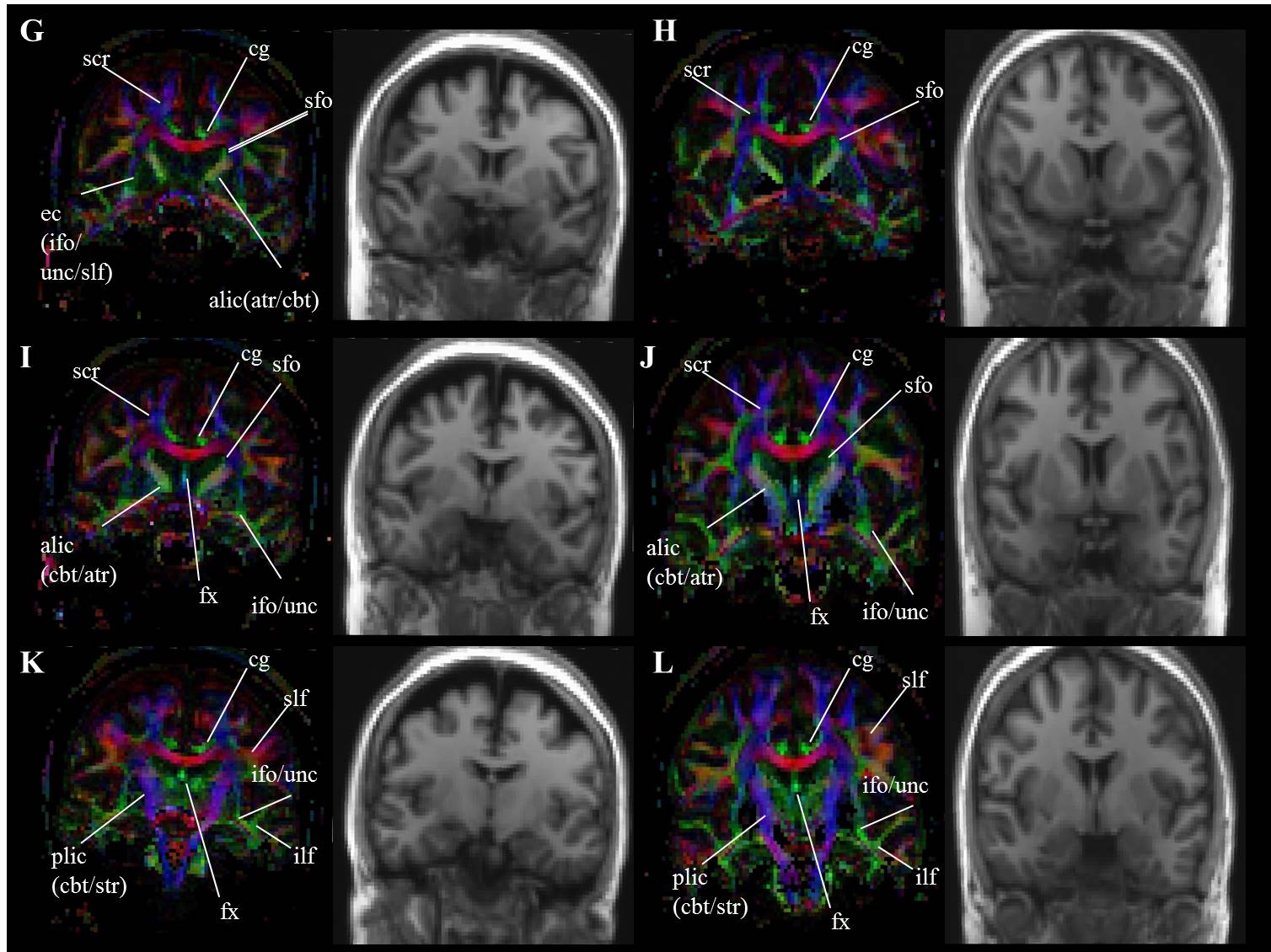

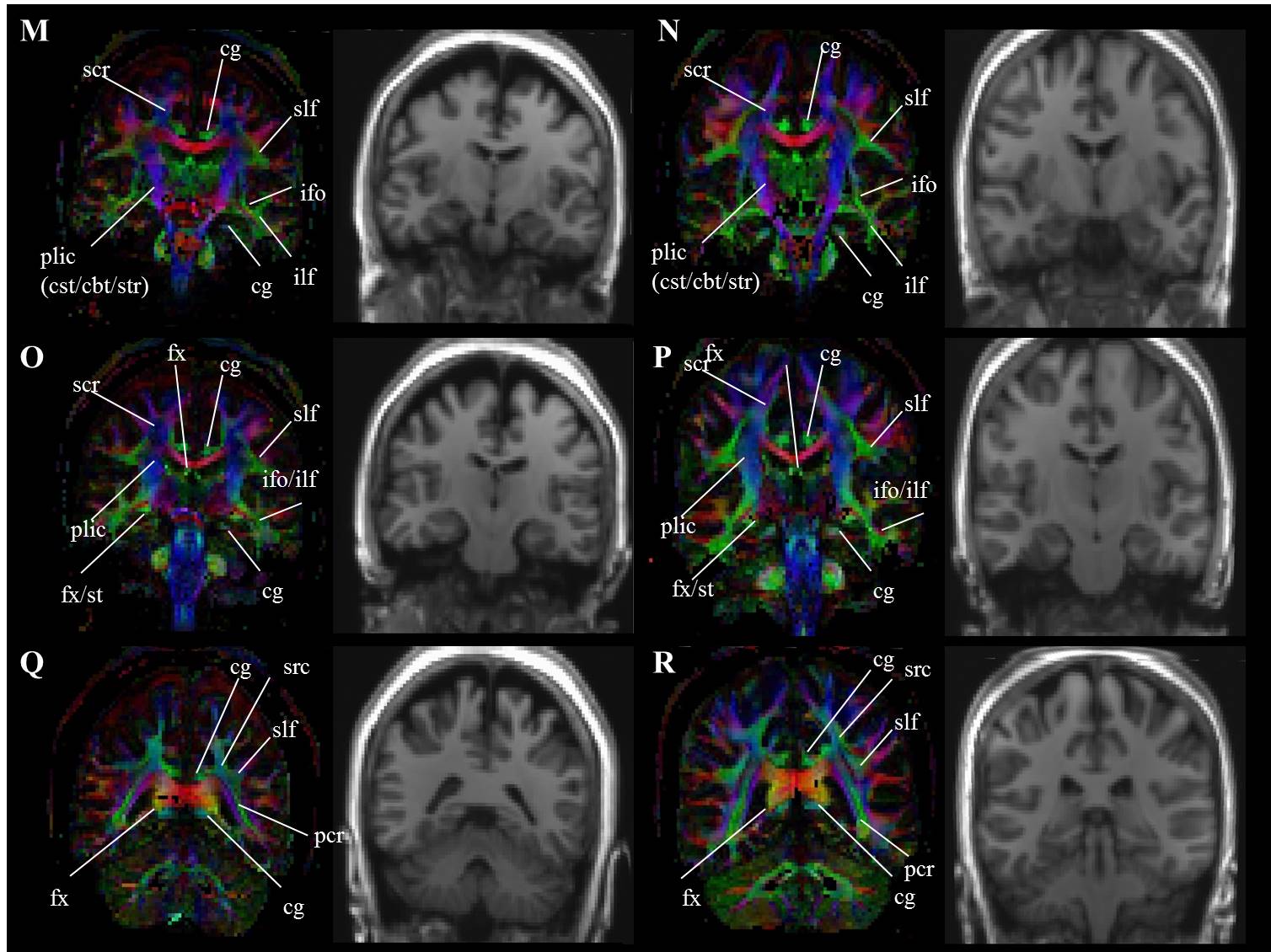

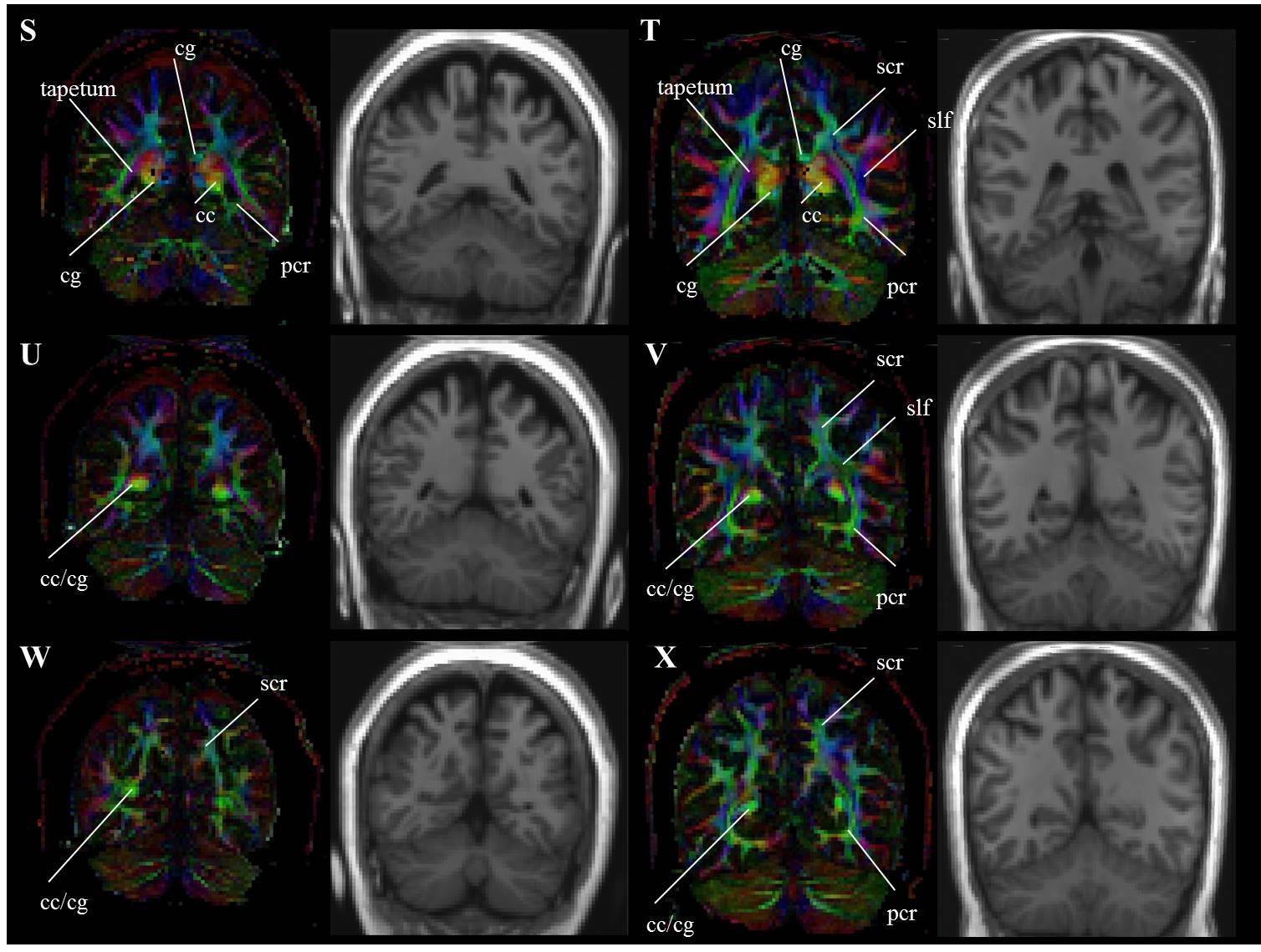


**Figure 2.** 2D coronal DT imaging color maps and their corresponding T1 background images showing projections of brain stem, projection and association fibers on patient’s (first and second columns) and a representative control (third and fourth columns) data.


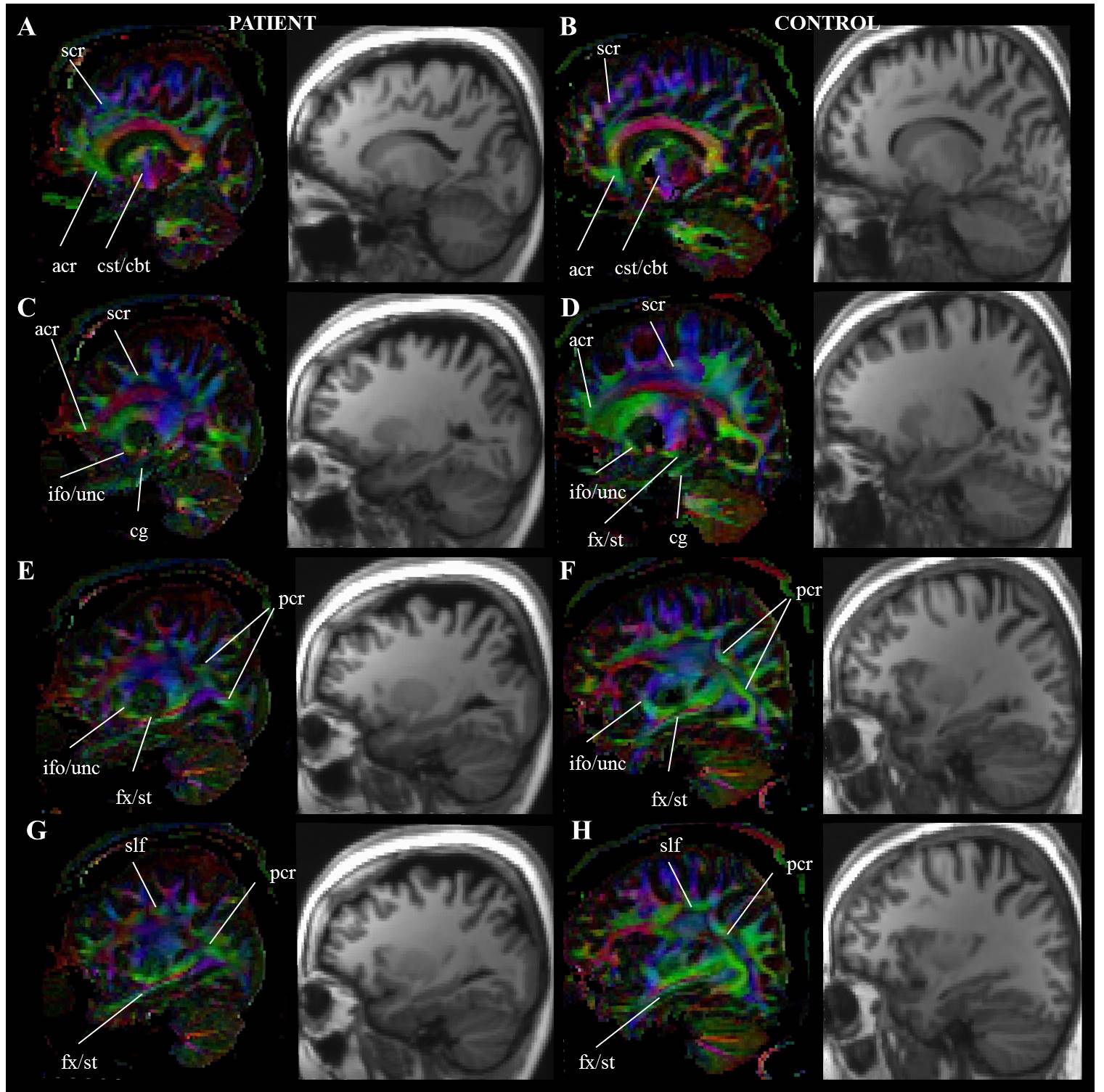


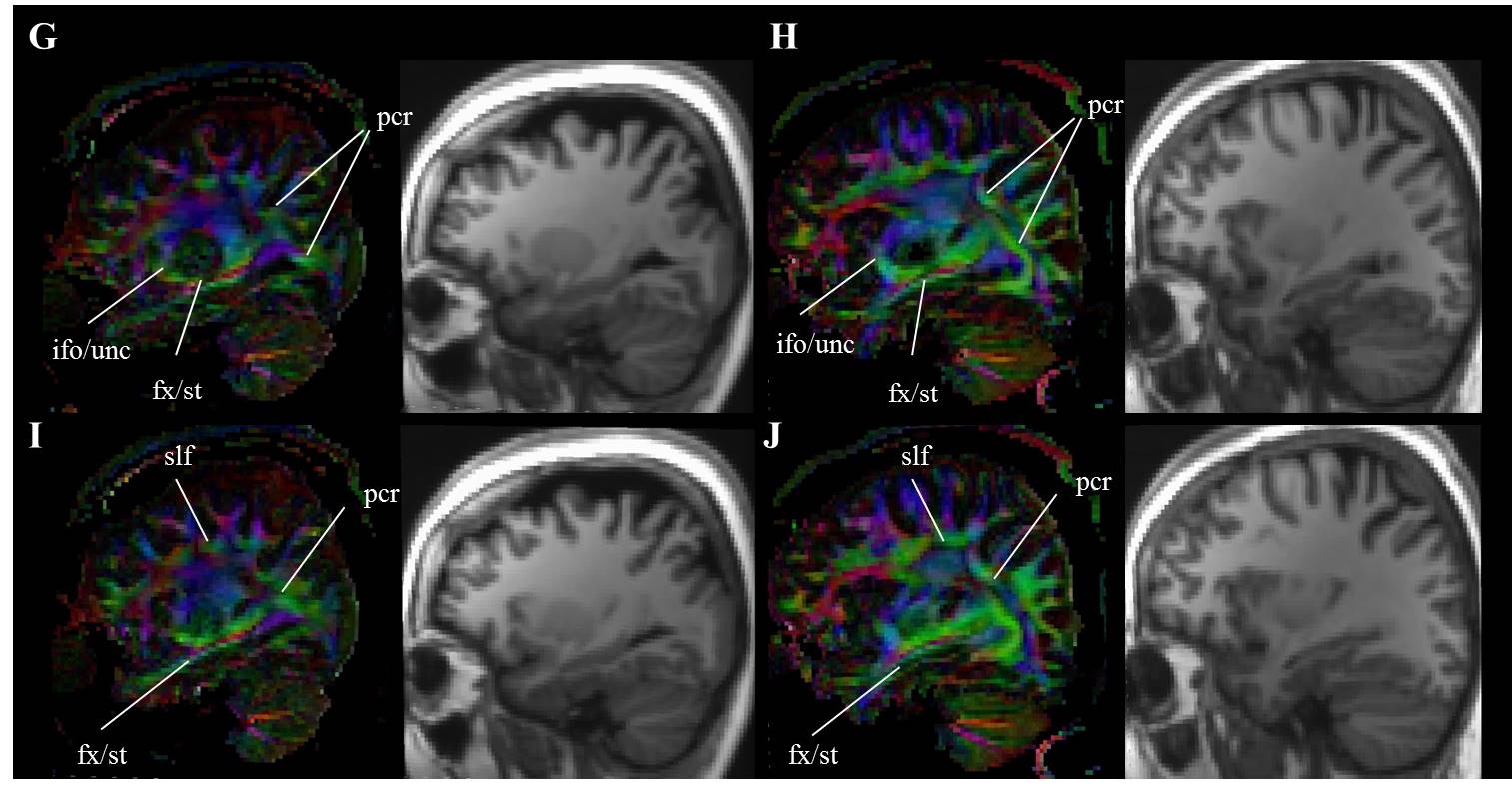
**Figure 3.** 2D sagital DT imaging color maps and their corresponding T1 background images showing projections of brain stem, projection and association fibers on patient’s (first and second columns) and a representative control (third and fourth columns) data.

**References**

Wakana, S., Jiang, H., Nagae-Poetscher, L. M., Van Zijl, P. C., Mori, S. (2004). Fiber tract–based atlas of human white matter anatomy. *Radiology*, 230(1), 77-87.
